# Biological management of coffee wilt disease (*Fusarium xylarioides*) using antagonistic *Trichoderma* isolates

**DOI:** 10.3389/fpls.2023.1113949

**Published:** 2023-03-17

**Authors:** Afrasa Mulatu, Negussie Megersa, Demelash Teferi, Tesfaye Alemu, Ramesh Raju Vetukuri

**Affiliations:** ^1^ Department of Microbial, Cellular and Molecular Biology, Addis Ababa University, Addis Ababa, Ethiopia; ^2^ Department of Biology, Bule Hora University, Bule Hora, Ethiopia; ^3^ Department of Chemistry, Addis Ababa University, Addis Ababa, Ethiopia; ^4^ Ethiopian Institute of Agricultural Research, Jimma Agricultural Research Center, Jimma, Ethiopia; ^5^ Department of Plant Breeding, Swedish University of Agricultural Sciences, Alnarp, Sweden

**Keywords:** bioassays, biocontrol agents, field evaluation, formulation, *Trichoderma* isolates

## Abstract

Coffee wilt disease (CWD) is a serious threat to the food security of small-scale farmers in Ethiopia, causing significant reductions in coffee yield. Currently, there are no effective control measures available against the causative agent of CWD, *Fusarium xylarioides*. The main objective of this study was therefore to develop, formulate, and evaluate a range of biofungicides against *F. xylarioides*, derived from *Trichoderma* species and tested under *in vitro*, greenhouse, and field conditions. In total, 175 *Trichoderma* isolates were screened as microbial biocontrol agents against *F. xylarioides*. The efficacy of two biofungicide formulations, wettable powder and water dispensable granules, were tested on the susceptible Geisha coffee variety in three different agro-ecological zones in southwestern Ethiopia over three years. The greenhouse experiments were set up using a complete block design, while in the field a randomized complete block design was used, with twice yearly applications of biofungicide. The test pathogen spore suspension was applied to the coffee seedlings by soil drenching, and the subsequent incidence and severity of CWD evaluated annually. The mycelial growth inhibition profiles of the *Trichoderma* isolates against *F. xylarioides* ranged from 44.5% to 84.8%. *In vitro* experiments revealed that *T. asperelloides* AU71, *T. asperellum* AU131 and *T. longibrachiatum* AU158 reduced the mycelial growth of *F. xylarioides* by over 80%. The greenhouse study indicated that wettable powder (WP) of *T. asperellum* AU131 had the highest biocontrol efficacy (84.3%), followed by *T. longibrachiatum* AU158 (77.9%) and *T. asperelloides* AU71 (71.2%); they also had a significant positive impact on plant growth. The pathogen-treated control plants had a disease severity index of 100% across all the field experiments, and of 76.7% in the greenhouse experiments. In comparison to untreated controls, the annual and cumulative disease incidence over the three years of the study period varied from 46.2 to 90%, 51.6 to 84.5%, and 58.2 to 91%, at the Teppi, Gera and Jimma field experimental locations. Overall, the greenhouse and field experiments and *in vitro* assays support the biocontrol potential of *Trichoderma* isolates, and *T. asperellum* AU131 and *T. longibrachiatum* AU158 in particular are recommended for the management of CWD under field conditions.

## 1 Introduction

East Africa is one of the most suitable regions for coffee production and export across the globe (International Coffee Organization (ICO), 2016). The center of origin and natural diversity of *Coffea arabica* L., the world’s most significant commercial coffee species, is in the Afromontane rainforests of southwestern Ethiopia, and Ethiopia has conducive temperature, rainfall, soil type, and pH levels at appropriate altitudes for successful coffee production (Alemu and Sokar, 2000; Woldetsadik and Kebede, 2000; Vega et al., 2009; Muleta et al., 2013; Nsabimana and Tirkaso, 2020). Coffee has played a key role for centuries in the Ethiopian economy, and represents the main cash crop cultivated by small-scale farmers, with social, economic, political and ecological benefits (Adugna et al., 2010; Jefuka et al., 2010; Getachew et al., 2012; Tefera et al., 2012). More than 25 million Ethiopians are engaged in the production, processing, and marketing of coffee crops, from which they derive a direct or indirect source of income (ICO, 2016). Overall, Ethiopia is Africa’s main exporter of coffee, but the sector is facing the combined threats of genetic erosion, market price fluctuations, climate change, displacement of coffee with other cash crops, and production constraints such as emerging fungal and bacterial diseases (Adugna et al., 2010; Avelino et al., 2011; Tsegaye, 2018).

One of the most economically significant diseases endangering Ethiopia’s Arabica coffee industry is coffee wilt disease (CWD), caused by the fungus *Fusarium xylarioides* (Adugna et al., 2010; Alemu, 2012; Getachew et al., 2012). *Fusarium xylarioides* is presumed to be a heterothallic ascomycete, with a natural sexual or teleomorphic state that produces fertile perithecia in dead coffee plants (Adugna et al., 2005). While the prevalence of CWD varies depending on the type of coffee production system (forest, semi-forest, garden, or plantation), Ethiopia’s overall 30–40% decline in coffee yield as a result of CWD presents a huge challenge for smallholder coffee producers. Coffee wilt disease is also threatening the global environmental benefit of Ethiopia’s coffee trees, which sequester over 200 million tons of carbon (Moat et al., 2017).

Current gaps in the development of management schedules for CWD include the lack of resistant coffee varieties, a lack of efficient chemical controls, a shortage of virulent biocontrol agents that may work at the field level, and an unclear mode of action. The ability of *F. xylarioides* to survive in the absence of coffee plants has substantial implications for managing the disease through chemical and cultural controls (Girma et al., 2009; Belachew et al., 2017a; Krishnan, 2017). The Ethiopian coffee industry is also being severely and negatively affected by climate change. According to Moat et al. (2017), depending on the emissions scenario, the bioclimatically suitable area for growing Arabica coffee could decrease 39–59% by the end of the 21st century. The rate of coffee plant death as a result of rapidly rising temperatures is alarming, particularly in the context of bacterial and fungal diseases (Moat et al., 2017). Additionally, the physiological and genetic variability of the pathogen is reducing the durability of resistance in coffee plants (Belete et al., 2014).

Ethiopia’s recent increase in CDW infections highlights the need for long-term integrated disease management (IDM) strategies. The evolution of fungicide-resistant strains of plant diseases, climate change and the growing global concern over chemical residues in the food chain have increased interest in developing biological solutions that are both effective and practical from both an economic and ecological standpoint. To date there are no effective control measures available for the management of CWD, but an eco-friendly and efficient biological solution is urgently required because of the destructiveness of the disease, the lack of resistant coffee cultivars, and the significance of coffee to the national economy.

*Trichoderma* species are already widely used to prevent plant pathogens and boost plant immunity in greenhouse and field conditions. However, while the potential of *Trichoderma* species for plant pathogen control is well recognized, this approach has been largely unexploited for many diseases of tropical perennial crops including coffee plants. The biocontrol potential of *Trichoderma* species is vast because of their beneficial interactions with plants *via* the production of cell wall-degrading enzymes (CWDEs), secondary metabolites, competition for nutrients and space, and antimicrobial compounds that are active against a wide range of plant pathogens, including *Fusarium* species (Saravanakumar et al., 2016b; Thambugala et al., 2020). *Trichoderma* species are found in Ethiopian coffee rhizosphere soils due to the diverse ecological features and climatic conditions of the country’s coffee-growing zones. Our previous study (Mulatu et al., 2022a) revealed that large *Trichoderma* species existed in native forest, semi-forest and garden coffee production systems, where CWD frequently found. Hence, using native and pathogen-specific microbial biocontrol agents (MBCAs) has the benefit of preventing a particular disease and improving the positive interaction between rhizosphere microbiome and plant immunity (Rao et al., 2016; Yu and Hochholdinger, 2018; Reghmit et al., 2021). However, there are only limited scientific studies on protecting coffee trees against *F. xylarioides* by using MBCAs belonging to the genus *Trichoderma*, under either greenhouse or field conditions. Thus, the application of a connventional biological control technique comprising the collection and release of possible *Trichoderma* species from the coffee arabica center of origin was examined in order to reduce the incidence and severity of CWD.

The development of new biocontrol agents to combat plant pathogens necessitates the screening of a large number of potential antagonists before formulating the agent in order to maximize its shelf life, facilitate delivery, and improve bioactivity (Montesinos, 2003; Kashyap et al., 2022; Manzar et al., 2022a; Mulatu et al., 2022a). A crucial element in the production of microbial biopesticides is the development of cutting-edge technologies for maximizing both quantity and quality. *Trichoderma* based formulation is considered as a stable and standardized combination of inert and active ingredients that produces a straightforward, secure, and highly effective solution to use against the target disease. Numerous formulation types, such as dusts, granules, pellets, wettable powders, capsules or beads, water-dispersible granules, or emulsifiable liquids, have been reported. Wettable powder (WP) and water dispensable granules (WDG) are two of these that are frequently used agricultural input formulations with good safety record. By assessing the effect of ingredients and processes on the physical characteristics, biological activity, storage stability, and field efficacy of selected biocontrol agents, formulation-based solutions to significant problems related to MBCA stability, efficacy, and toxicity have been addressed (Mulatu et al., 2021; Mulatu et al., 2022a; Mulatu et al., 2022b). Early screening processes have already evaluated coffee plants as a target crop, the CWD pathogen and the epidemiological stage of the disease, and the antagonistic efficacy of the proposed *Trichoderma* isolates, resulting in a pool of potential biocontrol candidates (Alemu and Kapoor, 2004; Tiru et al., 2013; Mulatu et al., 2022a; Manzar et al., 2022b).

To prove the value of biocontrol as a “best practice” for coffee farmers, the economic viability of optimal biocontrol systems was assessed. The study’s findings will significantly advance scientific understanding and provide useful information for making decisions. The planned research is helpful for reducing the effects of CWD and improving coffee production, which in turn improves Ethiopia’s ability to feed its people. The effective use and exploitation of these research findings will boost coffee crop yield as well as the nation’s foreign revenues. The findings can be applied to the development of decision-supporting tools to guarantee Ethiopia’s sustainable coffee producing system. It will contribute to safeguard the future of coffee production in Ethiopia and other East African countries, sustain a crucial source of income for resource-strapped farming communities, and stabilize the national economies by strengthening the ability of people battling the CWD. Therefore, the main objective of this study was to develop, formulate, and evaluate biofungicides against *F. xylarioides* from the pool of *Trichoderma* isolates, under *in vitro*, greenhouse, and field conditions. The efficacy of two biofungicide formulations, WDG and WP, were tested on the susceptible Geisha coffee variety in three different agro-ecological zones in southwestern Ethiopia over a period of three years.

## 2 Materials and methods

### 2.1 Biocontrol screening of *Trichoderma* isolates

#### 2.1.1 Dual culture assay

A total of 175 *Trichoderma* isolates, previously identified from major coffee-growing regions of Ethiopia (Mulatu et al., 2022a), was evaluated against *F. xylarioides* following the method of Dennis and Webster (1971). Briefly, mycelial discs (5 mm in diameter) from 7-day-old growing edges of *Trichoderma* and *F. xylarioides* were placed on opposite sides of a potato dextrose agar (PDA) Petri dish (6 cm away from each other). Three replications of culture plates were used; control plates had no *Trichoderma* discs. The plates were incubated at 25°C with a 12-h photoperiod for 10 days. Isolate growth was monitored every 48 h, to determine the percentage inhibition (PI) of the pathogen by each *Trichoderma* isolate, using Szekeres et al.’s (2005) formula:


$$\text{PI}=\left(\frac{\text{R}1-\text{R}2}{\text{R}1}\right)\times 100$$

where R1 denotes the control pathogen’s radius, and R2 denotes the antagonistic pathogen’s radius. Following Matarese et al. (2012), a standard regression growth curve was created by measuring the radius of the *F. xylarioides* colony in each plate as well as the effectiveness of the *Trichoderma* isolates in inhibiting the mycelial growth of *F. xylarioides* (Dennis and Webster, 1971). Using the criteria established by Soytong (1988), the antagonistic activity of *Trichoderma* isolates was classified as follows: >75% extremely high antagonistic activity; 61–75% high antagonistic activity; 51–60% moderate antagonistic activity; 50% low antagonistic activity. To evaluate the biocontrol potential of *Trichoderma* species, *F. xylarioides* (DSM No. 62457, strain: IMB 11646) was used as a test pathogen.

#### 2.1.2 Agar diffusion assay

Based on the *in vitro* antagonistic assay, 10 *Trichoderma* isolates, with a PI > 75%, were used for secondary metabolite extraction, as recommended by Saravanakumar et al. (2016a): *T. viride* AU23, *T. longibrachiatum* AU32, *T. koningiopsis* AU70, *T. asperelloides* AU71, *T. asperellum* AU97, *T. harzianum* AU105, *T. aethiopicum* AU106, *T. asperellum* AU131, *T. longibrachiatum* AU158 and *T. asperellum* AU171. In brief, 1 liter of potato dextrose broth (PDB) was injected with 1 × 10^7^ spores/ml *Trichoderma* isolates, which were then cultivated for 21 days at 28°C. The mycelia were homogenized by addition of ethyl acetate, centrifuged at 5000 rpm for 20 min and filtered to remove the insoluble particles from the solutions. The culture filtrates were concentrated using rotary evaporator at 40°C. The concentrated extracts were then dissolved in methanol so that they could be partially purified in a Sephadex LH-20 column. The chromatographic fractions from the Sephadex column were tested against *F. xylarioides* using an agar diffusion assay on King B medium (20 g peptone, 1.5 g K_2_HPO_4_, 1.5 g MgSO_4_.7H_2_O, 10 ml glycerol, and 15 g agar-agar in distilled water at pH 7; Bano and Musarrat, 2004; Hernández-Rodríguez et al., 2008; Dhodary et al., 2018).

Every bioassay was carried out in triplicate and contrasted with similarly made solvent controls. The diameters of the inhibition zones were measured, and the PI determined. The plates were photographed using a Canon digital camera, and “Fiji ImageJ” used to analyze the images (GNU General Public License), so that the following equation could be applied (Khan et al., 2021):


$$\text{ PI}=\frac{\left[\left(\text{T}-\text{I}\right)-\left(\text{C}-\text{I}\right)\right]}{\left(\text{T}-\text{I}\right)}\times 100$$

where I denotes the initial hole diameter (5 mm), T denotes the diameter of treatment inhibition zones, and C denotes the diameter of solvent control inhibition zones.

#### 2.1.3 Biocontrol-related enzymes

Based on the same dual culture results (PI >75%), the 10 *Trichoderma* isolates were tested for hydrolytic enzyme activity. The isolates were grown on 1/10^th^ strength PDB for 48 h in a rotary shaker at 150 revolutions per minute (rpm) and 28°C. To induce enzyme production, basal salts of the following compositions were added: MgSO_4_.7H_2_O (0.3 g), (NH_4_)_2_SO_4_ (3 g), KH_2_PO_4_ (2 g), agar (15 g). They were augmented with 1% (*w/v*) colloidal chitin (Rojas-Avelizapa et al., 1999; El-Sobky et al., 2019), 1% (*w/v*) CMC, 1% (*w/v*) gelatin, and 2.0% (*w/v*) laminarin), to detect the enzymes chitinase, cellulase, protease and β-(1–3) glucanase (Nagpure et al., 2014; Putri et al., 2022), respectively. The incubation was continued for 24 h, and the culture filtrate separated by filtration and centrifugation (at 5000 rpm, 4°C, for 10 min) and used as the enzyme source (Maria et al., 2005; Agrawal and Kotasthane, 2012; Saravanakumar et al., 2017). *Fusarium xylarioides* was then inoculated in the center of Petri dishes and the following equation used to calculate the percentage enzyme activity (after Saravanakumar et al., 2016b):


hydrolysis capacity(%)=diameter of clear zone(cm)/diameter of F. xylarioides colony(cm)

### 2.2 Characterization of *Trichoderma* isolates for their plant growth-promoting attributes

#### 2.2.1 Quantitative analysis of indole-3-acetic acid production

*Trichoderma* isolates were cultured in a rotary shaker for 4–5 days at 150 rpm and 25°C, in PDB supplemented with L-tryptophan (0.1g/L) (Bader et al., 2020). At the end of the incubation period, mycelia and debris were separated by filtration and centrifugation, respectively. After a 10-min incubation in the dark, Salkowski reagent (2% 0.5 M ferric chloride in 35% chloric acid) was added to 1 ml of the culture filtrate, so that a pink–red color could be seen. After 30 min, the optical density (OD) was assessed at 530 nm using a UV/VIS spectrophotometer (Mwajita, 2017; Kashyap et al., 2021). A calibration curve of standard IAA solutions was used to ascertain the IAA concentration, and an uninoculated PDB was maintained as a control. The experiment was carried out in triplicate, twice for each isolate.

#### 2.2.2 Siderophore production

Siderophore production by the *Trichoderma* isolates was determined using a modified chrome azurol S (CAS) agar test (Qi and Zhao, 2013). The isolates (5 mm) were inoculated onto CAS media and incubated for 5–7 days at 25°C. The development of a pink, yellow or orange color around or below the fungal colony indicated that the *Trichoderma* isolates had the ability to produce siderophores (Chen et al., 2019). Measurements were taken in triplicate.

### 2.3 *In planta* assay to evaluate bioefficacy against *F. xylarioides* under greenhouse and field conditions

#### 2.3.1 Experimental sites

From January 2016 to June 2021, experiments were conducted in three different agro-ecological zones of southwestern Ethiopia, at the Teppi, Jimma, and Gera agricultural research centers (**Supplementary Figure S1**). These locations are within Ethiopia’s main coffee-growing area, and have documented repeated CWD infestations (Belachew et al., 2015; Teferi and Belachew, 2015; Belachew et al., 2017b). Climatic information from January 2018 to December 2021 was provided by the National Meteorology Agency of Ethiopia (**Supplementary Table S1**). The greenhouse trials were conducted at the Jimma Agricultural Research Center (JARC).

#### 2.3.2 Preparation of *F. xylarioides* inoculum

Fresh coffee branches (twigs) were harvested from healthy trees and used to support the growth of *F. xylarioides* (Getachew et al., 2012; Mulatu et al., 2013; Tiru et al., 2013). The twigs were cut into small pieces (10 cm long) and lightly scraped to expose fresh wood. A Petri dish containing the twigs and a tiny roll of thoroughly wet cotton wool was sterilized in an autoclave. Each twig was then inoculated with 3 discs (5 mm) of *F. xylarioides* and incubated for 7–10 days at 22 ± 2°C (Adugna et al., 2005; Getachew et al., 2012). Twigs exhibiting healthy colony growth were completely rinsed off with sterile water to collect the conidia. The suspension was agitated with a magnetic stirrer and then filtered through two layers of cheese cloth. The spore concentration was adjusted with a hemocytometer (Neubauer chamber, Germany) to 2.3 × 10^6^ conidia/ml and kept at 4°C until seedling inoculation (Adugna, 2004; Getachew et al., 2012).

#### 2.3.3 Development and formulation of biofungicides

Two *Trichoderma*-based formulations, water dispensable granules (WDG) and wettable powder (WP), were used. To develop and formulate these products*, Trichoderma* isolates were cultured in PDB for 7–10 days, at 150 rpm and 25°C in an orbital shaker (Optima shaker OS-72, New Delhi, India). The broth was filtered *via* muslin cloth to eliminate the *Trichoderma* biomass, and the filtrate used to provide the active ingredients for the formulation of biofungicides (Mulatu et al., 2021; Vimala Devi et al., 2021). Talcum powder was used as the carrier substrate, glycerol as the dispersing agent, carboxymethyl cellulose (CMC) as the wetting agent, and a combination of white rice and wheat bran (1:2 *w/w*) as the bulking agent. WDG was developed using talcum powder as the carrier substrate and bulking agent, alginic acid as the dispersing agent, and CMC as the wetting agent (Jangir et al., 2021). The chemical reagents were selected according to the Collaborative International Pesticides Analytical Council’s (CIPAC) recommendations.

To formulate the WP, sterile talcum powder (500 g/kg) and dispersing agent (10 g/kg) were mixed with a substrate colonized by a *Trichoderma* isolate (440 g/kg), dried overnight, and then packed in polypropylene bags, sealed and kept at room temperature for later application in the greenhouse and field experiments (Ramanujam et al., 2010; Sachdev and Singh, 2016). To promote spore adhesion to the talcum powder, the pH of the *Trichoderma* colonized substrates was first brought to neutral by adding CaCO_3_ at a rate of 15 g/kg (Irfan et al., 2012).

To develop the WDG, the dispersion agent (10 g/kg), wetting agent (50 g/kg), and bulking agent (440 g/kg) were combined with adsorbed substrate (500 g/kg) (Jangir et al., 2021; Vimala Devi et al., 2021). After adding autoclaved water (15 to 20%), the product was mixed well, and the paste fed manually through an extruder machine using a 0.3-mm mesh screen. After air-drying overnight, the product was broken into granules and used for the greenhouse and field experiments. The spore concentration of the formulated biofungicides (WP and WDG) were adjusted to 10^9^ spores/ml for greenhouse and field applications.

#### 2.3.4 Bioefficacy of biofungicides under greenhouse conditions

Systematic pot culture experiments were carried out at JARC from January 2016 to April 2017. Normal sandy loam soil was taken from the coffee farm at JARC, passed through a 20-mm mesh, and sterilized in an autoclave at 121°C for 1 h. The experiments were performed in earthen plant pots with a capacity of 4 kg sandy loam soil (pH = 5.56, electronic conductivity (EC) 360 dS/m, organic carbon content (OC) 1.82%, and total nitrogen (TN) 0.45%; **Table S2**). A susceptible Geisha coffee variety was used to test the effectiveness of the variously prepared biofungicides (WDG and WP), and the following isolates were used for the treatments: *T. viride* AU23, *T. longibrachiatum* AU32, *T. koningiopsis* AU70, *T. asperelloides* AU71, *T. asperellum* AU97, *T. harzianum* AU105, *T. aethiopicum* AU106, *T. asperellum* AU131, *T. longibrachiatum* AU158, and *T. asperellum* AU171. *Fusarium xylarioides* alone was used as a positive control (PC), and no *F. xylarioides* or *Trichoderma* isolate was used as a negative control (NC). Before treatments were applied to the seedlings, the WDG product (5 g) was dissolved in water (20 ml) at room temperature for 24 h to initiate spore germination. Under greenhouse and field settings, control and biofungicides treatments were applied under the same conditions.

The treatments (control and biofungicides) were applied in a growth chamber at an optimum temperature of 22–25°C, and relative humidity (RH) above > 95%, to promote infection. After 10 days, the inoculated seedlings were moved and placed onto greenhouse benches. During the testing period, the greenhouse temperature fluctuated from 15 to 30°C, and the humidity between 65 and 85%. After 4 months, once two to three pairs of true leaves had developed, WDG was applied to the pots the following day by soil drenching at a concentration of 20 ml/seedling (Netsere et al., 2015). WP was applied to the pots by mixing the product (5 g/seedling) with the soil around the seedling rhizospheres. The seedlings were infected 7 days after WDG and WP treatment with 50 ml of the test pathogen’s spore suspension (2.3 × 10^6^ conidia/ml) by soil drenching (10 ml/seedling) (Adugna, 2004; Getachew et al., 2012; Jangir et al., 2021).

Throughout the duration of the experiment, sufficient water was applied to maintain the growth of the seedlings in the greenhouse. The experiments were replicated three times, and the treatments were assigned using a completely randomized design (CRD) (Tiru et al., 2013). To maintain adequate populations of the antagonists in the soils, and so favor biological control as proposed by Brown et al. (2019), WDG and WP were applied twice a year, once at the beginning of the trial (May 2016) and again after six months. In order to determine the effectiveness of WDG and WP in controlling CWD, disease assessment evaluations were monitored periodically after 3 months of treatment application. Morpho-agronomic characteristics were also recorded to evaluate the effect of the treatments on seedling growth.

#### 2.3.5 Bioefficacy of *Trichoderma* WP under field conditions

Based on the results of the greenhouse experiments, the WP from *T. longibrachiatum* AU32, *T. asperellum* AU71, *T asperellum* AU131, and *T. longibrachiatum* AU158 was chosen for the field experiments, which ran from January 2018 to September 2021 at Teppi, Jimma, and Gera agricultural research centers. Sick plots that had had a reported 50–100% incidence of CWD in previous years (Adugna et al., 2010; Getachew et al., 2012; Zeru et al., 2012; Belachew et al., 2015; Belachew et al., 2017b) were selected. The soil characteristics were a sandy loam, pH = 5.65, EC = 351.67 dS/m, and OC = 1.25% (**Table S2**). Four replicates of each treatment were used, in a randomized complete block design (RCBD). Each experimental site contained a total of 240 healthy Geisha coffee seedlings. In the first week of July 2018, six-month-old seedlings were transplanted into the field. Three months later, in the first week of October 2018, WP (10 g/seedling) was mixed into the soil surrounding the rhizosphere of the seedlings. A dual application of WP was made each year, the first in early October and the second in March (October 2018 to September 2021). Seven days after WP treatment, each seedling received 50 ml of pathogen spore suspension (10^6^ spore/ml), using the soil drenching method (Adugna et al., 2010). To keep the soil moist and promote germination of *Trichoderma* in the field, sufficient water and mulching was applied. Treatments without the test pathogen and bioagents were used as negative controls.

#### 2.3.6 Assessment of CWD under greenhouse and field conditions

In the greenhouse setting, the number of wilted seedlings exhibiting external symptoms was monitored and recorded periodically for one year, while in the field the plants were monitored for three consecutive years. Every two weeks following the commencement of disease, wilt symptoms were evaluated across all the exp erimental set-ups. The degree of vascular discoloration, and the presence and absence of internal symptoms, were examined on samples of seedlings without external symptoms. In order to reisolate the test pathogen, stem tissues from symptomatic and asymptomatic seedlings (n = 5) were plated on synthetic nutrient agar (SNA) media. In order to track the progression of CWD at the level of each pot, plot and seedling, the seedlings’ condition and symptoms were recorded periodically using an arbitrary 5-point scale of disease severity index (DSI), where 1 = no disease/symptoms, 2 = 1–25% curled leaves and stunted growth, 3 = 26–50% yellowing, leaf wilting and defoliation, 4 = 51–75% leaf necrosis, leaf wilting, and abscission, and 5 = 76–100% wilted leaves, defoliation, and total coffee plant death (Musoli et al., 2008; Adugna et al., 2010).

The formulae used by Deketelaere et al. (2017) and Di Marco et al. (2021) were adopted to determine yearly and cumulated disease incidence (DI, %), DSI (%), and biocontrol efficacy (BE, %):

a) annual CWD assessments


$$\text{DI }\left(\%\right)=\frac{\text{No. of infected seedlings }}{\text{Total number of seedlings}}\;\times 100$$

$$\text{DSI }\left(\%\right)=\;\frac{\text{Σ }\left(\text{No. of diseased seedlings in each disease scale }\times \text{ value of relative scale}\right)\;}{\text{Total number of inspected seedlings }\times \;5\;}\;\times 100$$

$$\text{BE }\left(\%\right)=\frac{\text{ Control of disease incidence }–\text{ Treatment disease incidence }\;}{\text{Control disease incidence}}\;\times \;100$$

b) cumulated CWD assessments


$$\text{DI }\left(\%\right)=\frac{\text{No. of infected seedlings in that year and previous year }}{\text{Total number of standing seedlings in previous year}}\;\times 100$$

$$\text{DSI}(\%)=\frac{\sum \left(\text{No. of seedlings in each disease scale in that year and previous year }\times \text{value of relative scale}\right)}{\text{Total number of inspected seedlings in previous year}\;\times \;5\;}\times 100$$

$$\text{BE }\left(\%\right)=\frac{\text{ Cumulated control disease incidence }–\text{Cumulated treatment disease incidence}\;}{\text{Cumulated control disease incidence}}\;\times \;100$$

The treatment DI was the incidence of disease in pathogen and biocontrol agent-inoculated seedlings, while the control DI was the incidence of disease in pathogen-only inoculated seedlings (Di Marco et al., 2021).

At the end of the greenhouse and field study experiments, for each year after treatment application, the growth of the coffee plants, using parameters such as plant height (cm) and girth (cm), was recorded and analyzed.

### 2.4 Statistical analysis

An analysis of variance (ANOVA) was used to evaluate the data from the *in vitro* bioassays, hydrolytic enzyme activity assays, and greenhouse and field experiments. Tukey’s honestly significant difference (HSD) test was used as a *post hoc* test, and the parameter values satisfied the requirements for normality and homogeneity of variances. All the ANOVA analyses were carried out using R Statistical Software (v4.1.2; R Core Team, 2021) running in RStudio 1.1, with the help of the packages “agricolae”, “smplot”, “supernova”, “tidyverse” “dplyr”, “reshape2”, “ggpubr”, “rstatix”, and “ggplot2” (https://CRAN.R-project.org). Differences were considered significant at *p*<0.05.

## 3 Results

### 3.1 Biocontrol screening of *Trichoderma* isolates

**Figure 1:**
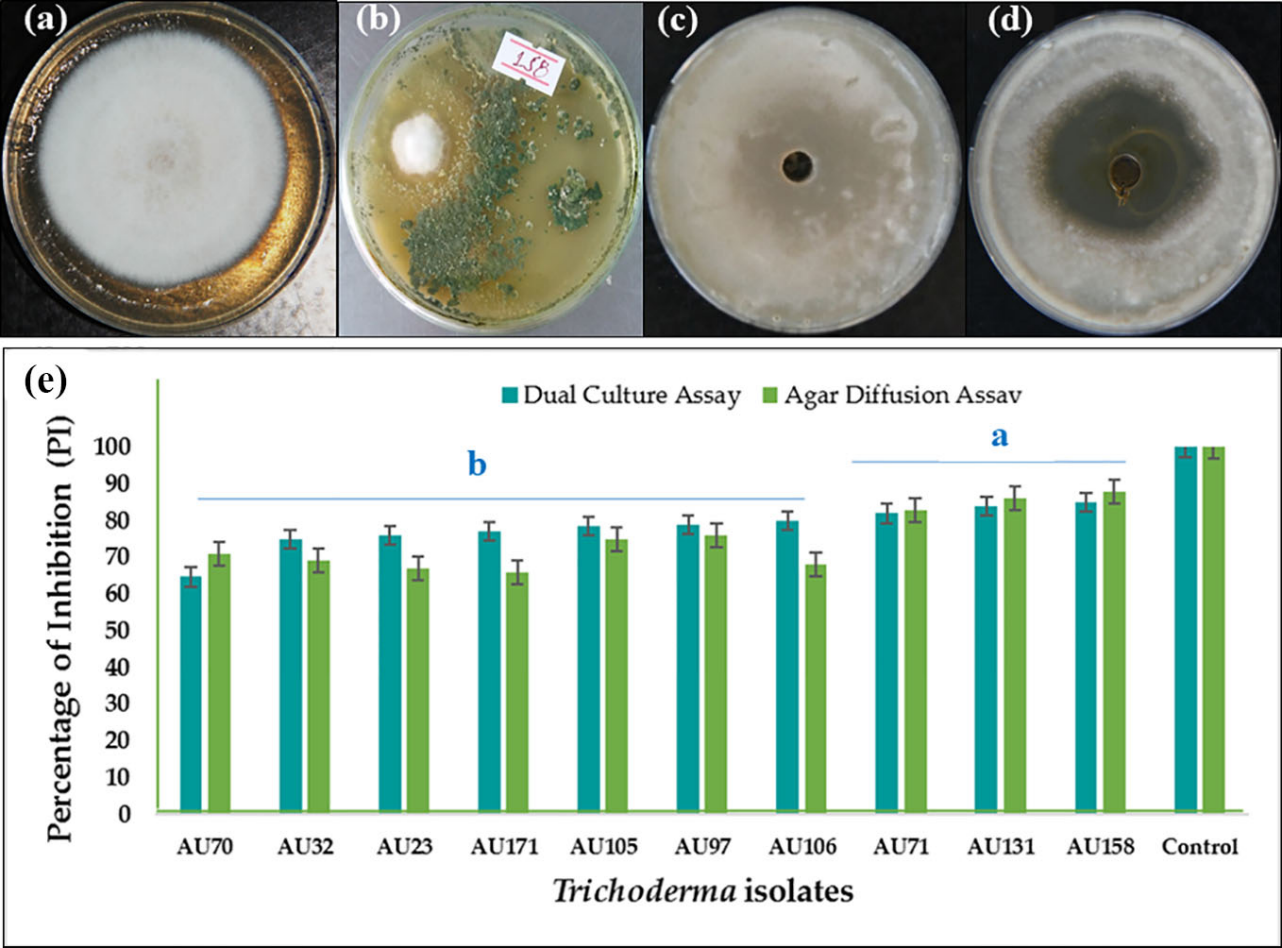
*In vitro* antagonistic activity of 10 *Trichoderma* isolates against *F*. *xylarioides* in dual culture and agar diffusion assays. **(A)** *F. xylarioides* control for dual culture assay, **(B)** *in vitro* dual culture assay showing inhibition of *F*. *xylarioides* by *T. longibrachiatum* AU158, **(C)** *F*. *xylarioides* control for agar diffusion assay, **(D)** agar diffusion assay showing inhibition of *F*. *xylarioides* by *T. longibrachiatum* AU158 and **(E)** bar graph for both dual culture and agar diffusion assay. Each value is the average of three duplicate samples with standard error, and various alphabets shown in superscript indicate mean treatments that are substantially different according to Tukey’s HSD posthoc test at p ≤ 0.05. Trichoderma isolates: *T. viride* AU23, *T. longibrachiatum* AU32, *T. koningiopsis* AU70, *T. asperelloides* AU71, *T. asperellum* AU97, *T. harzianum* AU105, *T. aethiopicum* AU106, *T. asperellum* AU131, *T. longibrachiatum* AU158 and *T. asperellum* AU171.

In the dual culture confrontation assays, *F. xylarioides* initially grew quickly, before stopping when it came into contact with the antagonist. All the *Trichoderma* isolates were able to inhibit the mycelial growth of *F. xylarioides* around the agar wells at the point of application. There was, however, significant differences between the isolates (*p<*0.05), with the percentage inhibition ranging from 44.5% to 84.8% (**Table S3**). Based on bioefficacy, 59 isolates showed moderate antagonistic activity, demonstrating between 51 and 60% inhibition of *F. xylarioides*, while another 96 isolates demonstrated high antagonistic activity, demonstrating 61–75% inhibition. Ten isolates demonstrated the highest levels of *in vitro* antagonistic activity against *F. xylarioides*, with mean inhibition values ranging from 75 to 84.8% (**Figure 1**). Extracts from *T. asperellum* AU71, *T. asperellum* AU131, and *T. longibrachiatum* AU158 displayed 83.5%, 86.7%, and 88.2% inhibition of *F. xylarioides*, respectively (**Figure 1**).

**Figure 2:**
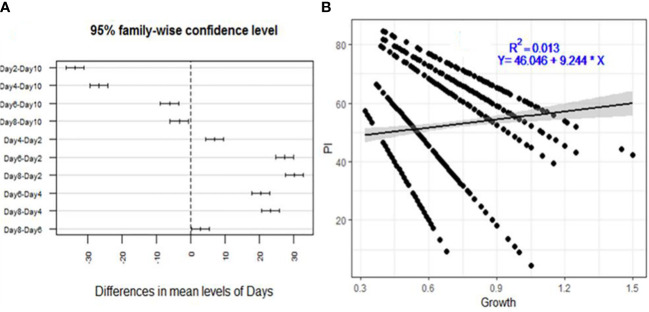
Descriptive analysis of percentage inhibition (PI) for the 175 *Trichoderma* isolates tested against *F*. *xylarioides*. **(A)** A pair-wise comparison of incubation period (days) and **(B)** Regression analysis between mycelial growth. PI, Percentage of Inhibition.

The inhibition of *F. xylarioides*’ radial growth in the agar diffusion assays was attributed to the production of secondary metabolites by each *Trichoderma* isolate, and the isolates had an impact on the pathogen’s development rate as early as the first day (**Figure 2A**). The levels of inhibition were negatively correlated with incubation duration (days), with a correlation coefficient (*R* ^2^) of 0.013 and a significance level of *p*<0.001 (**Figure 2B**).

### 3.2 Hydrolytic enzyme activity of *Trichoderma* isolates

**Figure 3:**
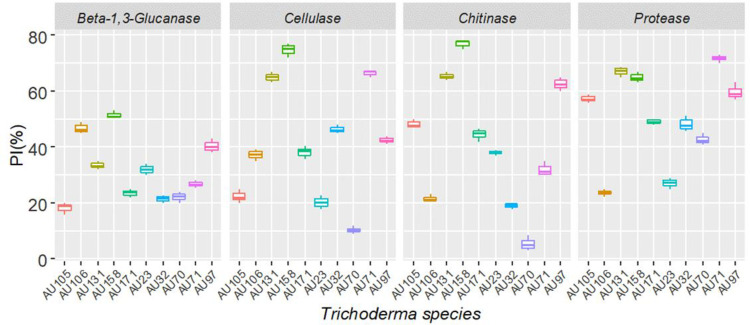
Boxplots of the hydrolytic enzyme activity of 10 *Trichoderma* isolates against *F. xylarioides. Trichoderma* isolates: *T. viride* AU23, *T. longibrachiatum* AU32, *T. koningiopsis* AU70, *T. asperelloides* AU71, *T. asperellum* AU97, *T. harzianum* AU105, *T. aethiopicum* AU106, *T. asperellum* AU131, *T. longibrachiatum* AU158 and *T. asperellum* AU171. The boundaries of the boxplots indicate the 25^th^ and 75^th^ percentiles, and the solid line within the boxes indicates the median value. PI, Percentage of Inhibition.

**Figure 4:**
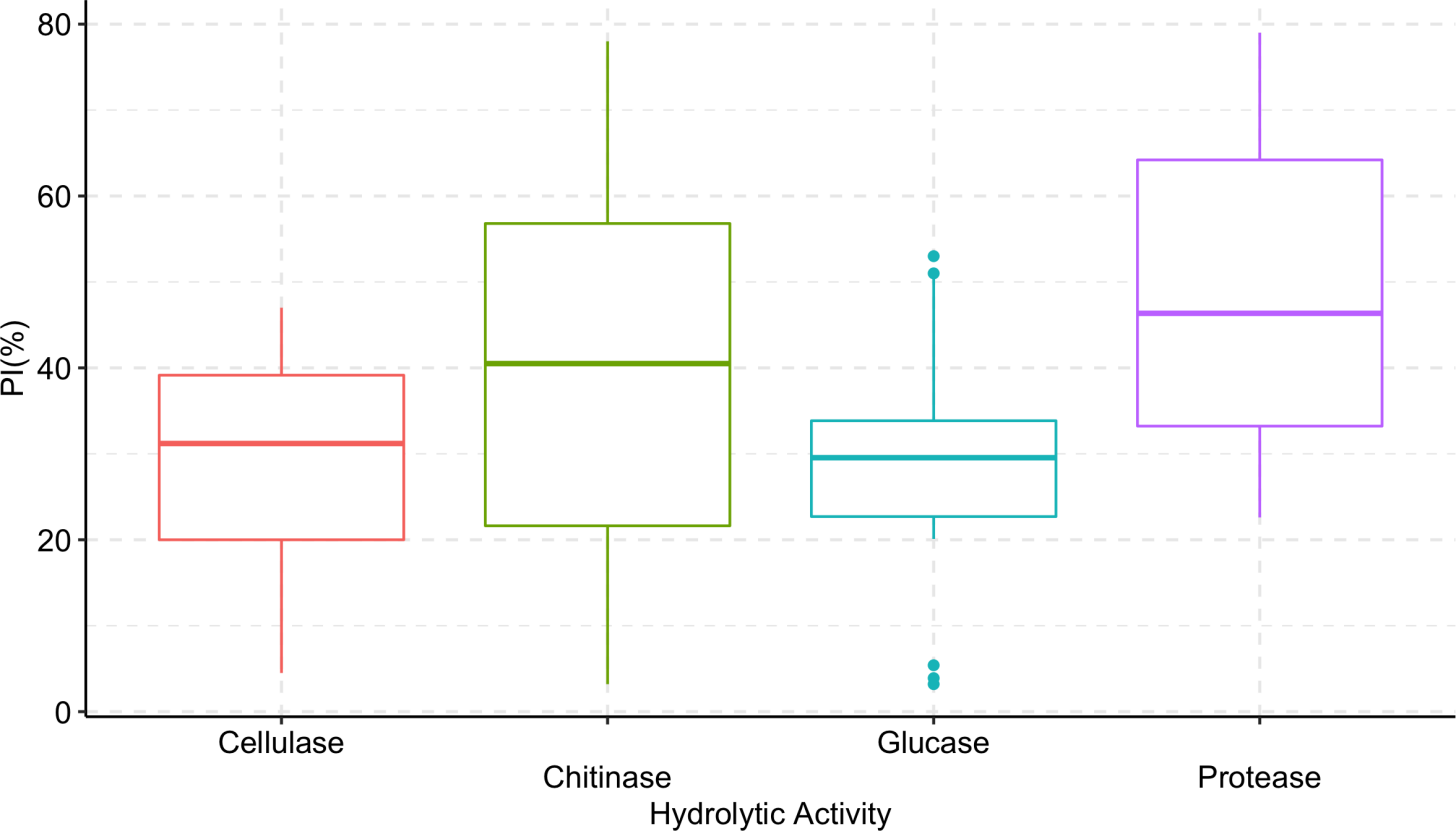
Boxplots showing the correlation between hydrolytic enzyme activity of *Trichoderma* isolates and percentage inhibition (PI) of *F. xylarioides* mycelial growth. The boundaries of the boxplots indicate the 25^th^ and 75^th^ percentiles, and the solid lines within the boxes indicate the median value. PI, Percentage of Inhibition.

*Trichoderma longibrachiatum* AU158 had higher enzyme activity levels for chitinase (76.9%), protease (64.9%), β-(1-3) glucanase (51.5%), and cellulase (44.9%) than the other isolates tested. *Trichoderma koningiopsis* AU70 had a low hydrolytic activity for chitinase (5.5%), while *T. aethiopicum* AU106 had a low protease activity (23.0%), *T. longibrachiatum* AU32 had a low cellulase activity (5.3%), and *T. harzianum* AU105 had a low β-(1-3) glucanase activity (4.2%) (**Figure 3**). There was a positive correlation between the hydrolytic and antagonist activity of the *Trichoderma* isolates (**Figure 4**). In general, chitinase, cellulase, and protease had moderate to high antagonistic activity against *F. xylarioides* mycelial growth, whereas β-(1-3) glucanase had low antagonistic activity (**Figure 4**).

### 3.3 Potential growth-promoting attributes of *Trichoderma* isolates

**Figure 5:**
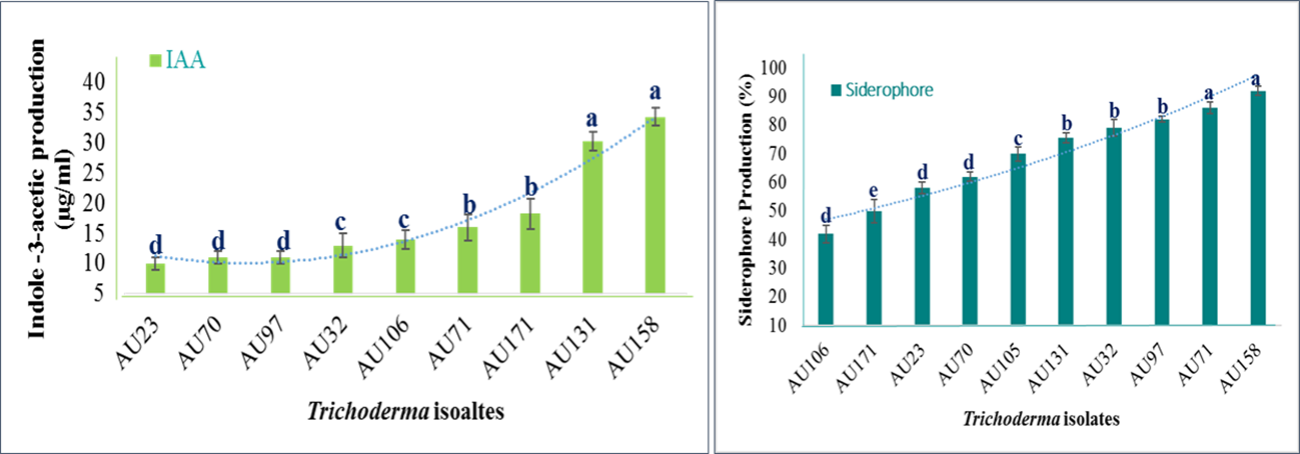
Quantitative estimates of the plant growth-promoting attributes of *Trichoderma* isolates *Trichoderma* isolates: *T. viride* AU23, *T. longibrachiatum* AU32, *T. koningiopsis* AU70, *T. asperelloides* AU71, *T. asperellum* AU97, *T. aethiopicum* AU106, *T. asperellum* AU131, *T. longibrachiatum* AU158 and *T. asperellum* AU171. Each value is the average of three duplicate samples with standard error, and various alphabets shown indicated mean treatments that are substantially different according to Tukey’s HSD posthoc test at p ≤ 0.05.

The plant growth-promoting attributes of *Trichoderma* differed significantly (*p*<0.05) between isolates. All of the isolates tested positive for IAA production, but *T. longibrachiatum* AU158, *T. asperellum* AU131, and *T. asperellum* AU171 displayed the highest levels (**Figure 5**), as quantified by spectrophotometry (**Figure S2**). The levels overall ranged from 8 to 44.5 μg/ml. All of the *Trichoderma* isolates were also able to synthesize siderophore units, with a range of 41.2 to 96.7%, as indicated by orange halos around the colonies on the blue CAS agar plate (**Figure S2**). The varied colors of the medium indicated that the synthesized siderophores differed structurally. After 6 days of incubation, *T. longibrachiatum* AU158, *T. asperelloides* AU71, and *T. asperellum* AU97 showed maximum siderophore units of 95.2%, 92.6%, and 84.2%, respectively. *Trichoderma aethiopicum* AU106 produced the fewest siderophore units (41%), while other *Trichoderma* isolates produced moderate levels of siderophore units, as shown in **Figure 5**.

### 3.4 Bioefficacy of biofungicides under greenhouse conditions

**Figure 6:**
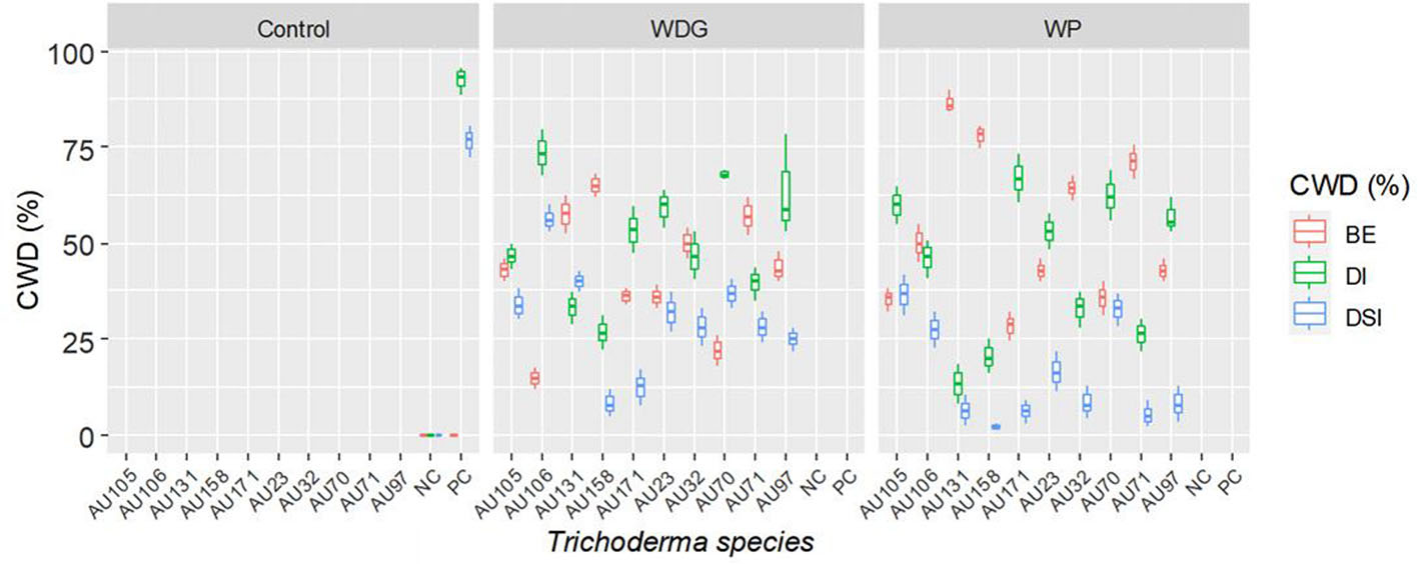
Boxplots of the antagonistic effectiveness of *Trichoderma* isolate WDG and WP products against *F. xylarioides* on coffee seedlings under greenhouse conditions. *Trichoderma* isolates: *T. viride* AU23, *T. longibrachiatum* AU32, *T. koningiopsis* AU70, *T. asperelloides* AU71, *T. asperellum* AU97, *T. harzianum* AU105, *T. aethiopicum* AU106, *T. asperellum* AU131, *T. longibrachiatum* AU158 and *T. asperellum* AU171. CWD, Coffee wilt disease; DI, Disease Incidence; DSI, Disease Severity Index; BE, Biocontrol Efficacy; PC, Positive control; NC, Negative control; WP, Wettable Powder and WDG , Water Dispensable Granules. The boundaries of the boxplots indicate the 25^th^ and 75^th^ percentiles, and the solid line within the boxes indicates the median value.

**Figure 7:**
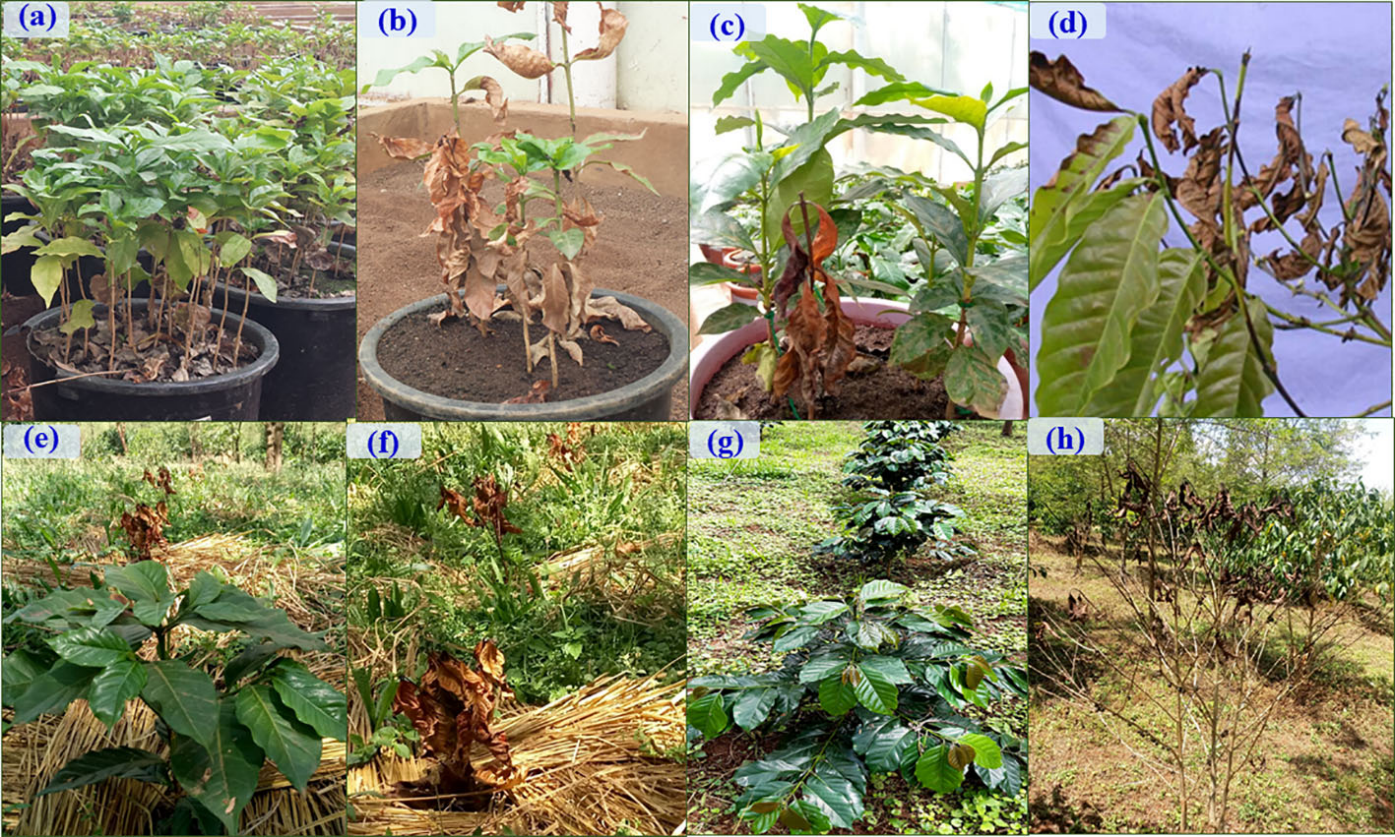
Characterization and evaluation of *Trichoderma* isolates as a biocontrol agent for against *F*. *xylarioides* on coffee seedlings under greenhouse and field conditions. Greenhouse experiment: **(A)** Negative control (*F. xylarioides* and biofungicides were not applied), **(B)** Positive control (*F. xylarioides* was applied to Geisha coffee seedlings) and **(C)** Geisha coffee seedlings treated with biofungicides (WDG and WP). Field experiment: **(E)** Negative control (*F. xylarioides* and biofungicides were not applied), **(F)** Positive control (*F. xylarioides* was applied to Geisha coffee plants) and **(G)** Geisha coffee seedlings treated with Wettable powder (WP) biofungicide. Typical symptoms: **(D)** and **(H)** are characteristic wilting symptoms manifested in Geisha coffee plants. The coffee plants treated with biofungicides and challenged with the pathogen showed healthy growth indicating the protection offered by *Trichoderma* species as a biocontrol agent.

Greenhouse experiments were conducted to evaluate the biocontrol efficiency of selected *Trichoderma* isolates against *F. xylarioides* using Geisha coffee seedlings. CWD infection was found in all replicates of pots treated with the pathogen. Seedlings treated with only the *F. xylarioides* spore suspension had the highest percentage incidence of CWD (92.5%) and DSI (76.7%) (**Figure 6**). After three months, typical partial wilting symptoms appeared in the positive control treatments (**Figure 7B**). However, compared to positive control treatments, the application of WDG and WP significantly (*p<*0.05) reduced the DI and DSI (**Figure 7C**). The DI of seedlings treated with *T. asperellum* AU131 WP was significantly reduced (15.7%), followed by *T. longibrachiatum* AU158 WP (22.1%). *Trichoderma asperellum* AU131 WP had the highest BE (84.3%) for reducing CWD, followed by *T. longibrachiatum* AU158 WP (77.9%) and *T. asperelloides* AU71 WP (71.2%) (**Figure 6**). The highest DI was observed in seedlings inoculated with *T. aethiopicum* AU106 (73.7%) WDG, *T. koningiopsis* AU70 WP (67.8%), *T. asperellum* AU97 WDG (63.4%), and *T. asperellum* AU171 WDG (53.5%). The highest DSI was observed with *T. aethiopicum* AU106 WDG (56.3%), while the lowest DSI was observed with *T. longibrachiatum* AU158 WP (8.3%). The distilled water-inoculated seedlings did not exhibit any disease symptoms (**Figure 7A**). In general, WP formulations caused a statistically significant reduction in CWD DI and DSI in the treated pots compared to WDG (**Figure 6**). As a result, WP-based formulations were chosen for the field experiments.

**Figure 8:**
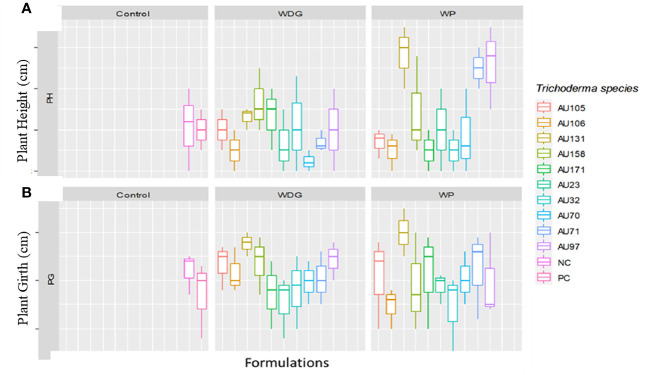
Boxplots of plant growth parameters for coffee seedlings treated with *Trichoderma* bioformulations. *Trichoderma* isolates: *T. viride* AU23, *T. longibrachiatum* AU32, *T. koningiopsis* AU70, *T. asperelloides* AU71, *T. asperellum* AU97, *T. harzianum* AU105, *T. aethiopicum* AU106, *T. asperellum* AU131, *T. longibrachiatum* AU158 and *T. asperellum* AU171. **(A)** Plant height and **(B)** plant girth. WP, Wettable Powder; WDG, Water Dispensable Granules; PC, Positive control and NC, Negative control. The boundaries of the boxplots indicate the 25^th^ and 75^th^ percentiles, and the solid line within the boxes indicates the median value.

Phenological indices revealed that seedlings treated with *Trichoderma* formulations grew significantly faster than untreated control seedlings (**Figure 8**). Overall, seedlings treated with *T. asperellum* AU97 and *T. asperellum* AU131 WP and WDG had higher plant heights and girths than untreated and *F. xylarioides*-treated seedlings. Thus, the use of WP and WDG formulations not only had an antifungal effect in controlling CWD, but also a growth-promoting effect (**Figure 8**).

### 3.5 Bioefficacy of *Trichoderma* WP under field conditions

**Figure 9:**
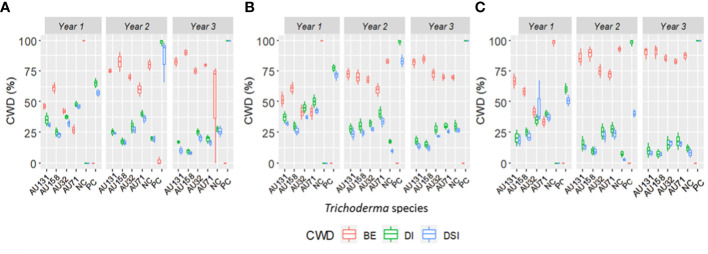
Boxplots representing the annual distribution of CWD for three consecutive years. **(A)** Teppi field site, **(B)** Gera field site and **(C)** Jimma field site. *Trichoderma* isolates: *T. longibrachiatum* AU32, *T. asperellum* AU71, *T. asperellum* AU131, *T. longibrachiatum* AU158. PC, Positive Control; NC, Negative Control; CWD, Coffee Wilt Disease; BE, Biocontrol Efficacy; DI, Disease Incidence and DSI, Disease Severity Index. Year 1 = 2018/19, Year 2 = 2019/20 and Year 3 = 2020/21. The boundaries of the boxplots indicate the 25^th^ and 75^th^ percentiles, and the solid line within the boxes indicates the median value.

WP formulations were applied to the susceptible Geisha coffee variety in the field to test the biocontrol potential of the chosen *Trichoderma* isolates in an ecologically relevant habitat. Six months after *F. xylarioides* application, the first symptoms of CWD were observed. The wilting began in the upper branches and spread to the entire tree canopy, resulting in a generalized green leaf defoliation (**Figures 7D, H**). Throughout the study period, all plants treated with *Trichoderma* isolates showed a significant reduction in DI and DSI compared to the positive controls (*p*<0.05) (**Figures 7F, G**, **9**). In the first (2018/19), second (2019/20), and third (2020/21) years, the percentage of DI in plants treated with *F. xylarioides* alone was 60.5, 98, and 100% at Jimma, 65, 96.5, and 100% at Teppi, and 77.3, 98.4 and 100% at Gera, respectively. The CWD DI and DSI was higher at Gera than at the other field sites. From the second year (2019/20), untreated negative control coffee plants also exhibited a lower DI and DSI (**Figure 7E**), as a result of the presence of natural inoculum in the field experimental sites (**Figure 9**).

A reduction in annual CWD DI and DSI was seen in all the experimental fields over the study period, although it varied significantly (*p*<0.05) from year to year (**Figure 9**). There were no significant differences in DI between the treatments in Gera and Teppi during the first year (2018/19), and the positive control had a significantly higher DI (**Figures 8A, B**). Over the three years, at all the experimental sites all plants treated with *Trichoderma* isolates showed a significant (*p*<0.05) reduction in DI and DSI compared to the positive controls. *Trichoderma asperellum* AU131 and *T. longibrachiatum* AU158 gave the best results, with a maximum BE at Gera of 81.8 and 84.5%, at Teppi of 82.5 and 90%, and at Jimma of 90 and 91%, respectively (**Figure 9**). The CWD pressure on the plants was reduced by all *Trichoderma* treatments, and the BE was different across all the field sites over the three years. However, only moderate disease reduction was seen with *T. asperellum* AU71 and *T. longibrachiatum* AU32 across all sites.

**Figure 10:**
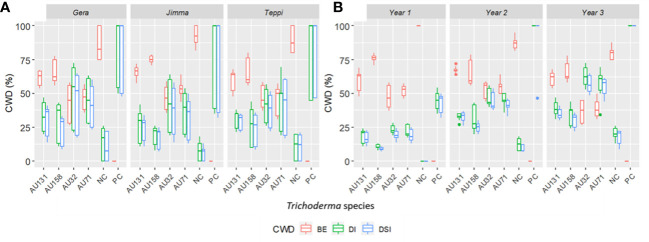
Boxplots showing cumulated incidence and severity of CWD, and biocontrol efficacy (BE) of *Trichoderma* formulations. **(A)** Effect of *Trichoderma* treatments against *F*. *xylarioides* at three field experimental sites and **(B)** Cumulated incidence and severity of CWD surveyed over three years. *Trichoderma* isolates: *T. longibrachiatum* AU32, *T. asperellum* AU71, *T. asperellum* AU131, *T. longibrachiatum* AU158. PC, Positive Control; NC, Negative Control; CWD, Coffee Wilt Disease; BE, Biocontrol Efficacy; DI, Disease Incidence and DSI, Disease Severity Index. Year 1 = 2018/19, Year 2 = 2019/20 and Year 3 = 2020/21. The boundaries of the boxplots indicate the 25^th^ and 75^th^ percentiles, and the solid line within the boxes indicates the median value.

**Figure 11:**
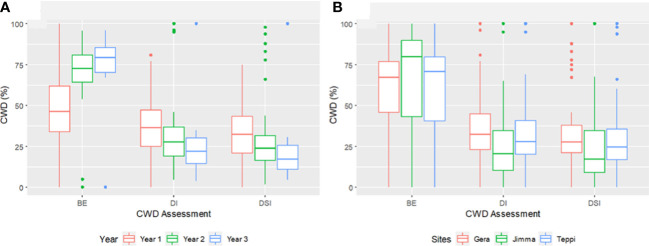
Boxplots summarizing the cumulated CWD assessments **(A)** across three years and **(B)** at different experimental field sites. CWD, Coffee Wilt Disease; BE, Biocontrol Efficacy; DI, Disease Incidence and DSI, Disease Severity Index. Year 1 = 2018/19, Year 2 = 2019/20 and Year 3 = 2020/21. The boundaries of the boxplot indicate the 25^th^ and 75^th^ percentiles, and the solid line within the boxes indicates the median value.

Overall, the three-year preventive applications demonstrated a very high reduction in CWD development, plant death, and cumulative incidence compared to untreated controls, with BE ranging from 46.2 to 90% at Teppi, 51.6 to 84.5% at Gera, and 58.2 to 91% at Jimma (**Figure 10**). There was a statistically significant reduction in the cumulative incidence of disease in the treated plots after just one year of treatment, but there was a difference in the level of disease reduction between the isolates. However, consistent BE effects were observed at all the sites over the three consecutive years (**Figures 10**, **11**). By the end of the third year, the cumulative BE of *Trichoderma* isolates was 67.7% at Gera, 71.4% at Teppi, and 80.5% at Jimma, respectively (**Figure 11B**). In the presence of natural inoculum of the test pathogen, the *Trichoderma* isolates appeared to provide efficient, sustainable, environmentally friendly, and long-lasting protection (**Figure 7F**).

**Figure 12:**
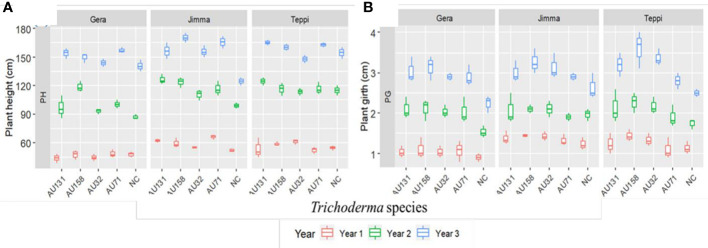
Boxplots of Geisha coffee plant growth after treatment with *Trichoderma* WP under field conditions. **(A)** Plant height and **(B)** Plant girth. *Trichoderma* isolates: *T. longibrachiatum* AU32, *T. asperellum* AU71, *T. asperellum* AU131, *T. longibrachiatum* AU158, NC, Negative Control; Year 1 = 2018/19, Year 2 = 2019/20 and Year 3 = 2020/21. The boundaries of the boxplot indicate the 25^th^ and 75^th^ percentiles, and the solid line within the boxes indicates the median value.

In general, a significant (*p*<0.05) promotion of plant growth was indicated by *Trichoderma* treatments under field conditions, compared to untreated controls (**Figure 12**). However, the annual plant height and girth values varied significantly (*p*<0.05) between individual *Trichoderma* isolates and experimental sites. At all sites, plants treated with *T. asperellum* AU71 and AU131, and *T. longibrachiatum* AU158, had higher plant height and girth values than untreated controls, while there was no significant difference in plant height or girth between the untreated controls and *T. longibrachiatum* AU32-treated plants (**Figure 12**).

## 4 Discussion

A large number of *Trichoderma* isolates from the rhizosphere of *C. arabica* trees have been screened (Mulatu et al., 2022a) and evaluated under *in vitro*, greenhouse, and field conditions with *F. xylarioides*, in order to identify potential native biocontrol agents. As a result of the stepwise multiple screening, several isolates have emerged as viable candidates for biocontrol agents in the management of CWD. All of the candidate isolates displayed strong antagonistic activity, and successfully inhibited the mycelial growth of *F. xylarioides*, with mean PIs ranging from 44.4 to 84.8% (**Table S3**). However, there was significant variation across the isolates in the strength of the antagonistic response, which could have been the result of various factors.

According to Filizola et al. (2019), some species prevent the growth of phytopathogens by hyper-parasitism, while others achieve it through antibiosis or competition for nutrients and space. *Trichoderma* species have been shown to proliferate more quickly than competing phytopathogens as a result of their more efficient use of food sources (Marik et al., 2019). Additionally, harzianolide, peptaibols, pyrones, and other secondary metabolites generated by *Trichoderma* species have been described as having antimicrobial potential and acting as plant growth promoters (Hermosa et al., 2014; Manzar et al., 2021; Zhang et al., 2021). The number of extracellular lytic enzymes and secondary metabolites produced by each species of *Trichoderma* work in concert to promote a synergistic effect of their many mechanisms of action (Hermosa et al., 2014).

*Trichoderma* species are known to produce a broad spectrum of bioactive secondary metabolites that can affect a wide range of phytopathogens, including *Alternaria alternata*, *Botrytis cinerea*, *Fusarium* species, *Pythium* species, *Rhizoctonia solani*, *Sclerotinia sclerotiorum*, and *Ustilago maydis* (Hyder et al., 2017; Sood et al., 2020). A number of commercial *Trichoderma*-based biopesticides have already been developed, and *T. asperellum, T. asperelloides, T. harzianum, T. longibrachiatum*, and *T. viride* are known to be effective against a variety of *Fusarium* species (Kari et al., 2012; Temesgen Belayneh et al., 2013; Błaszczyk et al., 2017; Mulatu et al., 2022a). Several studies have also demonstrated the effectiveness of *T. asperellum, T. asperelloides*, and *T. longibrachiatum* against different pathogens using a variety of modes of action, including antibiosis, mycoparasitism, competition for nutrients, and inducing resistance (Wu et al., 2017; Shrestha, 2019; Wei et al., 2019). Compared to pathogenic microbes, the ability of *Trichoderma* species to proliferate quickly offers them a notable advantage in the competition for nutrients, space, and dominance over their prey host, which are all equally significant and interconnected (Kari et al., 2012).

In the present study, *in vitro* experiments identified the 10 *Trichoderma* isolates with the most significant PI of the test pathogen. It appeared that the production of bioactive secondary metabolites, cell wall-degrading enzymes, IAA, and siderophores gave the isolates their antifungal properties, and the variation in their mycelial growth inhibition rates suggested that their antagonistic activity had various modes of action. In order for antibiotics or antifungal compounds to enter plant pathogens, hydrolytic enzymes are necessary (Strakowska et al., 2014; Saravanakumar et al., 2016b), and the isolates used in this study were able to produce chitinase, cellulase, protease, and β-(1,3)-glucanase. Previous studies indicating that chitinases are involved in triggering a plant’s defense mechanisms corroborate the current results (Baek et al., 1999; Gueye et al., 2020). The current results are also supported by studies that have found that the hydrolytic enzymes (cellulase, chitinase, protease, and β-(1,3)-glucanases) produced by *T. asperellum*, *T. harzianum*, and *T. longibrachiatum* have a high level of antagonistic activity against *F. oxysporum* (Schirmböck et al., 1994; Putri et al., 2022). These enzymes play a key role in the breakdown of the pathogen’s cell walls, which facilitates simple invasion and colonization by the biocontrol agents, induces a defense response and slows the spread of the pathogen while also indirectly promoting plant growth and development (Qualhato et al., 2013; Cherkupally et al., 2017). Overall, *T. asperellum* AU131 and *T. longibrachiatum* AU158 isolates appear to be the most promising biological control agents against *F. xylarioides*, primarily because of the synergistic effect of various mechanisms of action, including mycoparasitism, competition, production of bioactive secondary metabolites, and induction of systematic resistance (Aita et al., 2019; Mulatu et al., 2022b).

All the *Trichoderma* isolates tested led to a considerable reduction in CWD and promoted the growth of coffee seedlings under greenhouse conditions. *Trichoderma asperellum* AU131 WP displayed the best BE (84.3%) for reducing CWD, followed by *T. longibrachiatum* AU158 (77.9%) and *T. asperelloides* AU71 (71.2%). Similar findings have been seen with the application of a rhizosphere *Trichoderma* species under greenhouse conditions, which led to a 100% reduction in *Fusarium* wilt disease and boosted plant growth metrics by 250% (Thangavelu and Gopi, 2015). The current results demonstrate that *Trichoderma* isolates can promote the growth of coffee plants, which may be related to their capacity to produce IAA and siderophores, and engage in biocontrol activities by secreting hydrolytic enzymes. This supports the work of Singh et al. (2013), who discovered that the production of siderophore and hydrolytic enzymes inhibited a number of plant diseases, and elsewhere *Trichoderma* species have been shown to produce siderophores and IAA, and promote plant growth (Saber et al., 2017; Bader et al., 2020).

The experimental field sites were chosen based on their edaphic and climatic characteristics, crop history and level of *F. xylarioides* inoculum in soil, in order to evaluate selected formulations in a variety of real-life scenarios within a coffee-growing region. A previous study has shown that *T. asperellum, T. asperelloides*, and *T. longibrachiatum* are the three most dominant and prevalent *Trichoderma* species in Ethiopia’s major coffee-growing regions (Mulatu et al., 2022a), and the adaptability and versatility of *Trichoderma* species enables them to display antimicrobial activity under a wide range of climatic and environmental conditions. The current field study provides a fresh perspective on how these species could function as preventative biocontrol agents. At Gera, Teppi, and Jimma, the mean cumulative BE demonstrated by *Trichoderma* isolates for 2020/21 was 67.7%, 71.4%, and 80.5%, respectively. The lower efficacy at Gera could be explained by the levels of natural soil pathogen inoculum, which were much higher than at Jimma and Teppi. Additionally, the high altitude and low temperatures, below 15°C, at Gera are favorable for pathogen growth (**Table S1**). However, from the first year of field studies, the effectiveness of each *Trichoderma* isolate as a biocontrol agent was evident. The *Trichoderma* treatments show good and cumulative efficacy in reducing disease pressure over time, giving rise to the theory that a decline in annual incidence may be associated with improved plant defensive responses, as proposed by Di Marco et al. (2021). These findings are encouraging because they suggest that *Trichoderma* species can be used in the coffee ecosystem under various agro-climatic conditions.

The spread of CWD is aided by agronomic practices such as replanting, slashing, and close spacing, as well as transporting diseased trees from one location to another (Adugna et al., 2010; Tsegaye, 2018). As a result, affected coffee plants are typically uprooted and burned to control the spread of the disease. However, a socioeconomic survey revealed that 60% of Ethiopian farmers use the wood for fences, 26% build dwellings and animal shelters with the wood, 10% give their neighbors any leftover wilting trees for firewood, and 2% actually sell the trees (Adugna et al., 2010). The incidence of CWD is negatively impacted by these agronomic methods, with rates in garden and plantation coffee plots being noticeably higher. The repeated cycles of infection increased CWD’s effects on farmers. Coffee farming is no longer a reliable source of revenue for many farmers, who had lost their livelihoods. The fact that coffee prices were declining on international markets only made things worse. But for many, CWD had the most apparent direct impact. It was difficult to afford the inputs required to manage plots and try to recover output due to lower incomes. Di Marco et al. (2021) suggest that the best way to reduce the damage caused by grapevine leaf stripe disease is to apply formulated products to pruning wounds early and regularly. Di Marco et al. (2021) also reported on preventive applications of *T. asperellum* strain ICC 012 and *T. gamsii* strain ICC 080 against diseases of grapevine trunk (Esca Complex) over 9 years. A large reduction in symptom development was seen in the treated vines, as well as a reduction in annual and cumulative incidence and vine death, with overall disease reduction ranging from 66 to almost 90%. Young coffee plants, that have not yet been affected by the disease, should therefore be the perfect target for preventative protection against CWD, by using *Trichoderma*-based biofungicides before the occurrence and spread of the disease. The current field results suggest that it is crucial to apply *Trichoderma* WP as soon as the coffee seedlings are transplanted, in order to boost the effectiveness of the biocontrol agents and provide long-term protection.

Thangavelu and Gopi (2015) have shown that *Fusarium* wilt disease is significantly reduced by a combined application of rhizosphere *Trichoderma* species NRCB under field conditions, 15 days prior to planting, and 2 and 4 months after planting. This indicates that prolonged and repeated application of MBCAs in the field is necessary for continued effectiveness. A protocol of applying treatments twice a year to protect coffee plants from CWD is therefore suggested, based on the sanitary measures used in the current study. Coffee growers require IDM of CWD using coffee varieties that have some resistance, and disease treatments for both old and new plantings as a means of good sanitary measures. The field investigations unequivocally demonstrated that dual therapy applications were effective during Ethiopia’s short rainy seasons from September to October and February to May. However, in comparison to field treatments, applying biofungicides at the nursery stage may be more advantageous from a practical, financial, and efficiency point of view. In order to manage CWD effectively, realistic management techniques such as biocontrol need to eliminate microsclerotia or stop their germination in soil (Tesfaye et al., 2020). This strategy can only be achieved in field conditions by applying MBCAs to the planting holes, to inhibit microsclerotia viability around the rhizosphere and protect the coffee plants during the first year of planting (Flood et al., 2016).

Many researchers have reported that *Trichoderma* species are antagonistic against a variety of phytopathogens in dual culture tests, but not so far in the field, largely because field conditions are significantly different from *in vitro* conditions (Zamanizadeh et al., 2011; Kari et al., 2012). However, the present study has revealed *T. asperellum* AU131 and *T. longibrachiatum* AU158 to be promising biocontrol agents against *F. xylarioides*, based on a stepwise protocol from *in vitro*, to greenhouse, to field experiments. The WP formulation consistently reduced the DI and DSI of CWD and enhanced the growth of susceptible Geisha coffee plants in three different agro-climatic zones and years. These findings demonstrate that *Trichoderma* isolates can provide preventative protection for the management of CWD. The ability of *T. asperellum* AU131 and *T. longibrachiatum* AU158 to inhibit the mycelial growth of *F. xylarioides* under *in vitro*, greenhouse, and field conditions, as well as to improve the growth of coffee plants, means that they offer the most potential for reducing CWD DI and DSI, and this study provides a practical basis for the development of eco-friendly treatments and a useful methodology for developing preventative tools within an IDM strategy against CWD.

Soil samples from Ethiopia’s coffee rhizosphere have previously been used to examine the biodiversity and distribution status of *Trichoderma* species (Mulatu et al., 2022a). A variety of parameters, such as the type of coffee production system, pH, water availability, temperature, altitude, pesticides, and other biotic and abiotic stress conditions, appear to influence the distribution and ecological preferences of *Trichoderma* species. The three dominant species in Ethiopia’s coffee ecosystem, *T. asperellum, T. asperelloides*, and *T. longibrachiatum*, can be found in all of the country’s major coffee-growing regions and districts (Mulatu et al., 2022a). The data presented here have generated the hypothesis that the coexistence of *T. asperellum* AU131 and *T. longibrachiatum* AU158 in coffee plant rhizospheres may retard or even prevent the development of CWD. Furthermore, field testing of *T. asperellum* AU131 and *T. longibrachiatum* AU158 WP in sick plots in a hot-spot area clearly demonstrated that the pathogen and disease could be successfully controlled and managed, as evidenced by the decreased incidence and severity of disease. To the best of our knowledge, this is the first report of the technological development of *T. asperellum* AU131 and *T. longibrachiatum* AU158 from the laboratory to the field, resulting in effective application of a biocontrol-based management strategy for CWD in Ethiopia.

The secondary metabolites from these two *Trichoderma* isolates have been profiled, characterized, and elucidated using gas chromatography (GC-MS) and untargeted liquid chromatography-high resolution mass spectrometry (LC-HRMS), and their toxicological effects investigated using Swiss albino mice (Mulatu et al., 2022b). GC-MS identified the substances in the ethyl acetate extracts as alcohols, benzene acids, sesquiterpenes, phenols, ketones, and aromatic chemicals. At various concentrations, neither the examined spore suspensions nor the methanol extracts of these isolates were harmful or pathogenic to Swiss albino mice (Mulatu et al., 2022b). As the *Trichoderma* formulations developed here promote the biological control of the plant pathogen for long-term CWD management, *T. asperellum* AU131 and *T. longibrachiatum* AU158 are recommended for use by small-and large-scale coffee producers.

In-depth study of the molecular mechanisms governing plant-microbe interactions is essential for the development of novel bio-formulations since it may reveal interacting mechanisms involved in the rhizosphere (Vishwakarma et al., 2020). The molecular elements that control plant-*Trichoderma* interactions in perennial crops like coffee plants are little known, despite the significance of these interactions. Additionally, research into the mechanism of action against *F. xylarioides* as well as the population dynamics of *Trichoderma* species under field circumstances following treatment is ongoing. To determine the potential mechanisms and how *Trichoderma* species are behaving in the field, these areas of the study have been initiated and will be thoroughly explored. It is also being studied how the genes for the defense enzymes expressed by *Trichoderma* isolates function.

## 5 Conclusions

Based on their mycelial growth inhibition profiles, the two isolates with the highest levels of *in vitro* antagonistic activity against *F. xylarioides* were *T. asperellum* AU131 and *T. longibrachiatum* AU158. The WP of *T. asperellum* AU131 demonstrated the greatest reduction in CWD incidence (84.3%), followed by the WP of *T. longibrachiatum* AU158 (77.9%), and both also had a significant positive effect on coffee plant growth under greenhouse and field conditions. In comparison to untreated controls, the preventive applications of WP of both MBCAs over a three-year period led to a significant decrease in annual and cumulative DI, with the BE ranging from 46.2 to 90% at Teppi, 51.6 to 84.5% at Gera, and 58.2 to 91% at Jimma. After 3 years, the cumulative BE of *Trichoderma* isolates was 67.7% at Gera, 71.4% at Teppi, and 80.5% at Jimma.

To the best of our knowledge, this is the first report of a biological solution, taken from the laboratory into the field, that can effectively manage CWD. The significant reduction in DI and DSI observed over three years clearly demonstrates the preventive outcome of treating the coffee plant’s rhizosphere. This study also clearly demonstrates how a preventive approach can be used to control CWD within natural ecosystems. Disease pressure was significantly reduced across all the experimental sites, highlighting the critical role of bioformulated biofungicides in CWD management. The biocontrol potential of *T. asperellum* AU131 and *T. longibrachiatum* AU158, as well as their exudates, is supported by *in vitro* tests, and greenhouse and field studies. Small- and large-scale coffee farmers are advised to use these two bioagents in the treatment of CWD.

## Data availability statement

The original contributions presented in the study are included in the article/**Supplementary Material**. Further inquiries can be directed to the corresponding author.

## Author contributions

The study was conceptualized, designed, planned, and carried out by AM, TA, RV and DT. AM, TA, DT, NM, and RV analyzed, validated, and curated the data. AM wrote and prepared the original manuscript. TA, DT, NM, and RV reviewed and edited the manuscript. TA and RV handled the funding’s acquisition and administration. All authors read and agreed to the published version of the manuscript. All authors contributed to the article and approved the submitted version.
