# Supplementary material for: Biological management of coffee wilt disease (Fusarium xylarioides) using antagonistic Trichoderma isolates
Source: Front Plant Sci. 2023 Mar 17;14:1113949. doi: 10.3389/fpls.2023.1113949 (PMC10064059; doi:10.3389/fpls.2023.1113949)
Supplement: Supplementary file 1 [file DataSheet_1.docx]

**Supplementary Figures**


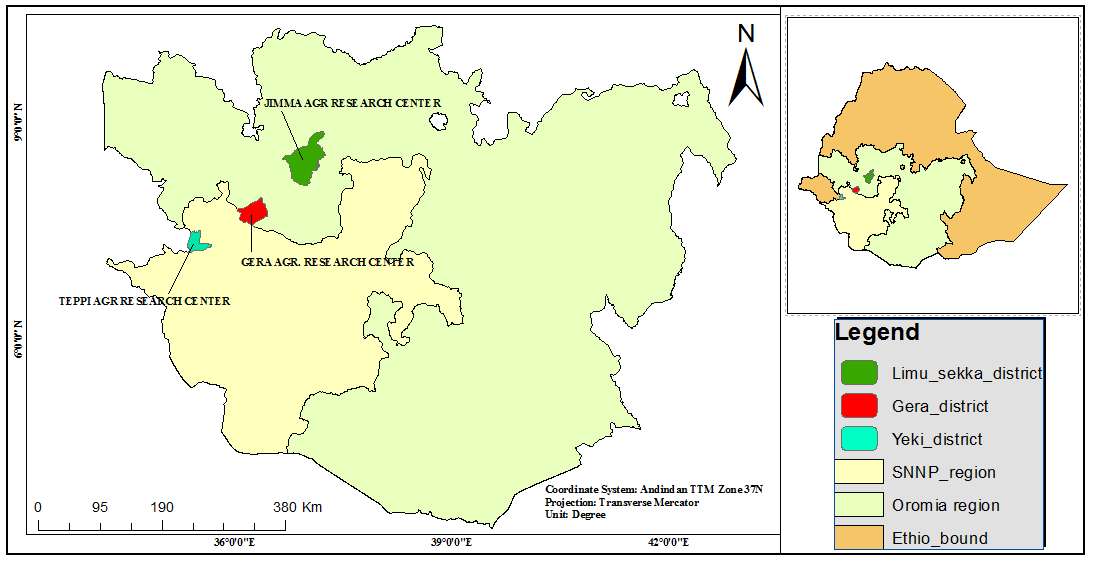


**Figure S1 | Map of field experimental sites, southwestern Ethiopia. SNNP = South Nations Nationalities Peoples.**


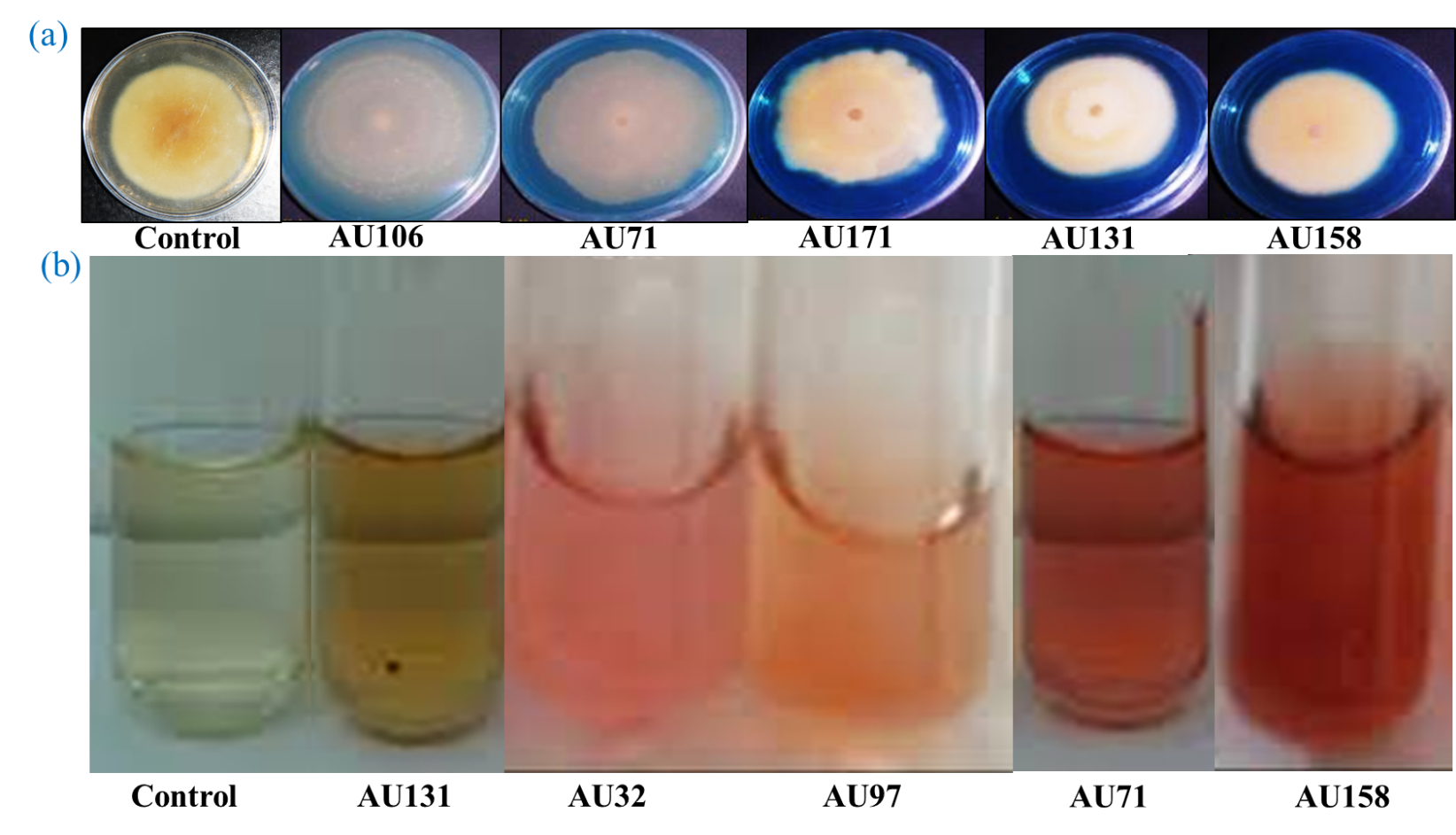


**Figure S2 | Quantitative estimates of the plant growth-promoting attributes of *Trichoderma* isolates.** (a) Siderophore production and (b) Indole-3- acetic acid production. *Trichoderma* isolates: *T. longibrachiatum* AU32, *T. asperelloides* AU71, *T. koningiopsis* AU70, *T. asperellum* AU97, *T. harzianum* AU105, *T. aethiopicum* AU106, *T. asperellum* AU131, *T. longibrachiatum* AU158 and *T. asperellum* AU171, Control = *Fusarium xylarioides* grown on potato dextrose agar (PDA).

**Supplementary Tables**

**Table S1 |** Meteorological data of the 2018/19- 2020/21 field experiments obtained from National Meteorology Agency of Ethiopia.

| Field sites | Altitude (m) | Coordinates | | Average Annual Temperature (^O^C) | | | | Average annual Rainfall (mm) | | | | |
| --- | --- | --- | --- | --- | --- | --- | --- | --- | --- | --- | --- | --- |
|  |  | Latitude (N) | Longitude (E) | 2018 | 2019 | 2020 | 2021 | 2018 | 2019 | 2020 | 2021 |  |
| TARC | 1200 | 7.1967° | 35.4289° | 20^a^/30^b^ | 18^a^/27^b^ | 22^a^/30^b^ | 17^a^/31^b^ | 1630 | 1652 | 1625 | 1570 |  |
| JARC | 1750 | 7.6739° | 36.8358° | 15^a^/27^b^ | 14^a^/29^b^ | 18^a^/30^b^ | 12^a^/27^b^ | 1557 | 1640 | 1665 | 1586 |  |
| GARC | 2050 | 50.8851° | 12.0807° | 9^a^/28^b^ | 12^a^/25^b^ | 10^a^/27^b^ | 14^a^/29^b^ | 1480 | 1472 | 1520 | 1468 |  |

Data (average *±* standard deviation) according to National Meteorology Agency of Ethiopia (Jimma, Agaro and Teppi meteorological station data). TARC = Teppi Agriculture Research Centre, JARC = Jimma Agriculture Research Centre and GARC = Gera Agriculture Research Centre. ^a^ minimum temperature and ^b^ maximum temperature.

**Table S2 |** **Physico-chemical properties of soil samples from field experimental sites**

| Sites | pH | Electrical Conductivity (dS/m) | Organic Carbon (%) | Total Nitrogen (%) | Soil  color | Soil  Texture |
| --- | --- | --- | --- | --- | --- | --- |
| TARC | 5.63 | 318 | 0.56 | 0.33 | Reddish brown | Sandy loam |
| JARC | 5.68  (5.56)* | 367  (360)* | 1.86  (1.82)* | 0.43  (0.45)* | Reddish brown  (Reddish brown)* | Sandy loam  (Sandy loam)* |
| GARC | 5.65 | 370 | 1.34 | 0.46 | Reddish brown | Sandy loam |
| Mean | 5.65 | 351.67 | 1.25 | 0.41 |  |  |

***** Soil Physico-chemical properties used for greenhouse experiment.

**Table S3 |** Identification, origin and *in vitro* antagonist activity of coffee rhizosphere derived *Trichoderma* isolates against *Fusarium xylarioides* on the tenth day (The mean percentage of inhibition of pathogen in dual culture, degrees of freedom-3). The top ten highly potential *Trichoderma* isolates (PI > 75%) were highlighted as **BOLD**. Accession numbers (tef1-α) was deposited in National Center for Biotechnology Information (NCBI).

| Taxa | Isolate ID | Accession Number (*tef1-α)* | District (Woreda) | Zone | Coffee Ecosystem | PI |
| --- | --- | --- | --- | --- | --- | --- |
| *T. asperellum* | AU1 |  | Gera | Jimma | Semi forest | 58 |
| *T. hamatum* | AU2 | MZ361591 | Gera | Jimma | Semi forest | 74.7 |
| *T. asperellum* | AU3 | MZ361592 | Gera | Jimma | Semi forest | 73.2 |
| *T. citrinoviride* | AU4 |  | Gera | Jimma | Semi forest | 68.8 |
| *T. asperelloides* | AU5 |  | Gera | Jimma | Semi forest | 72 |
| *T. reesei* | AU6 | MZ361593 | Melko | Jimma | Semi forest | 74 |
| *T. koningiopsis* | AU7 |  | Gera | Jimma | Semi forest | 60 |
| *T. asperellum* | AU8 | MZ361594 | Gera | Jimma | Semi forest | 74 |
| *T. longibrachiatum* | AU9 | MZ361595 | Yeki | Jimma | Semi forest | 70 |
| *T. aethiopicum* | AU10 | MZ361596 | Gera | Jimma | Semi forest | 62 |
| *T. asperelloides* | AU11 | MZ361597 | Gera | Jimma | Semi forest | 68 |
| *T. aethiopicum* | AU12 |  | Gera | Jimma | Semi forest | 64 |
| *T. asperellum* | AU13 | MZ361598 | Gera | Jimma | Semi forest | 56 |
| *T. longibrachiatum* | AU14 | MZ361599 | Yeki | Shaka | Semi forest | 74 |
| *T. asperellum* | AU15 | MZ361600 | Gera | Jimma | Semi forest | 58.8 |
| *T. harzianum* | AU16 |  | Gera | Jimma | Semi forest | 64 |
| *T. asperellum* | AU17 |  | Gera | Jimma | Semi forest | 52 |
| *T. asperellum* | AU18 |  | Gera | Jimma | Semi forest | 68 |
| *T. citrinoviride* | AU19 | MZ361601 | Gera | Jimma | Semi forest | 69.6 |
| *T. asperellum* | AU20 |  | Limmu Saka | Jimma | Semi forest | 72 |
| *T. asperellum* | AU21 | MZ361602 | Gera | Jimma | Semi forest | 64 |
| *T. asperellum* | AU22 | MZ361603 | Yeki | Shaka | Semi forest | 64 |
| *T. hamatum* | **AU23** | **MZ361604** | **Gera** | **Jimma** | **Semi forest** | **76.9** |
| *T. orientale* | AU24 | MZ361605 | Odo Shakiso | West Guji | Garden Coffee | 50 |
| *T. asperellum* | AU25 |  | Odo Shakiso | West Guji | Garden Coffee | 58.4 |
| *T. asperellum* | AU26 | MZ361606 | Odo Shakiso | West Guji | Garden Coffee | 58 |
| *T. asperellum* | AU27 |  | Odo Shakiso | West Guji | Garden Coffee | 58 |
| *T. asperelloides* | AU28 | MZ361607 | Gera | Jimma | Garden Coffee | 74 |
| *T. asperelloides* | AU29 | MZ361608 | Gera | Jimma | Garden Coffee | 64.8 |
| *T. hamatum* | AU30 | MZ361609 | Shebedino | Sidama | Garden Coffee | 62.8 |
| *T. asperellum* | AU31 |  | Shebedino | Sidama | Garden Coffee | 56 |
| *T. longibrachiatum* | **AU32** | **MZ361610** | **Gera** | **Jimma** | **Garden Coffee** | **75.2** |
| *T. asperellum* | AU33 |  | Shebedino | Sidama | Garden Coffee | 54 |
| *T. asperelloides* | AU34 | MZ361611 | Yeki | Shaka | Forest | 66.8 |
| *T. asperellum* | AU35 |  | Yeki | Shaka | Forest | 68 |
| *T. longibrachiatum* | AU36 |  | Melko | Jimma | Forest | 50 |
| *T. erinaceum* | AU37 | MZ361612 | Gera | Jimma | Forest | 50 |
| *T. asperellum* | AU38 | MZ361613 | Yeki | Shaka | Forest | 72 |
| *T. asperellum* | AU39 | MZ361614 | Gimbo | Kaffa | Forest | 60 |
| *T. longibrachiatum* | AU40 | MZ361615 | Gomma | Jimma | Forest | 44.5 |
| *T. brevicompactum* | AU41 | MZ361615 | Yeki | Shaka | Forest | 64 |
| *T. asperellum* | AU42 | MZ361615 | Gomma | Jimma | Forest | 51.6 |
| *T. asperellum* | AU43 |  | Haru | West Wollega | Forest | 45 |
| *T. asperellum* | AU44 | MZ361618 | Gomma | Jimma | Forest | 62 |
| *T. asperelloides* | AU45 |  | Gomma | Jimma | Forest | 73 |
| *T. asperellum* | AU46 | MZ361619 | Gomma | Jimma | Forest | 48 |
| *T. asperelloides* | AU47 | MZ361620 | Chena | Kaffa | Forest | 57.2 |
| *T. koningiopsis* | AU48 |  | Yeki | Shaka | Forest | 74 |
| *T. longibrachiatum* | AU49 | MZ361621 | Andaracha | Shaka | Forest | 47 |
| *T. asperellum* | AU50 | MZ361622 | Andaracha | Shaka | Forest | 52 |
| *T. hamatum* | AU51 | MZ361623 | Andaracha | Shaka | Forest | 72.8 |
| *T. asperellum* | AU52 |  | Andaracha | Shaka | Forest | 57.2 |
| *T. asperellum* | AU53 | MZ361624 | Andaracha | Shaka | Forest | 72 |
| *T. asperellum* | AU54 |  | Mena | Jimma | Semi forest | 58 |
| *T. asperelloides* | AU55 | MZ361625 | Mena | Jimma | Semi forest | 49 |
| *Non identified* | AU56 |  | Gewata | Kaffa | Forest | 66 |
| *T. asperelloides* | AU57 |  | Mena | Jimma | Semi forest | 56 |
| *T. erinaceum* | AU58 | MZ361626 | Gewata | Kaffa | Forest | 68 |
| *T. bissettii* | AU59 | MZ361627 | Aleta Wondo | Sidama | Garden Coffee | 58.8 |
| *T. longibrachiatum* | AU60 |  | Aleta Wondo | Sidama | Garden Coffee | 58 |
| *T. asperelloides* | AU61 | MZ361628 | Gomma | Kaffa | Garden Coffee | 52 |
| *T. asperellum* | AU62 |  | Jarso | West Wollega | Garden Coffee | 52 |
| *T. asperellum* | AU63 |  | Jarso | West Wollega | Garden Coffee | 70 |
| *Non identified* | AU64 |  | Yirga cheffe | Gedeo | Garden Coffee | 72 |
| *Non identified* | AU65 |  | Yirga cheffe | Gedeo | Garden Coffee | 44.8 |
| *Non identified* | AU66 |  | Yirga cheffe | Gedeo | Garden Coffee | 62.8 |
| *T. longibrachiatum* | AU67 |  | Aleta Wondo | Sidama | Garden Coffee | 64.8 |
| *T. asperellum* | AU68 |  | Yirga cheffe | Gedeo | Garden Coffee | 58 |
| *T. asperellum* | AU69 | MZ361629 | Wonago | Gedeo | Garden Coffee | 70 |
| *T. koningiopsis* | **AU70** | **MZ361630** | **Wonago** | **Gedeo** | **Garden Coffee** | **76** |
| *T. asperelloides* | **AU71** | **MZ361631** | **Yirga cheffe** | **Sidama** | **Semi forest** | **81.8** |
| *T. longibrachiatum* | AU72 | MZ361632 | Yirga cheffe | Gedeo | Semi forest | 60 |
| *T. asperellum* | AU73 | MZ361633 | Gewata | Kaffa | Semi forest | 64 |
| *T. asperellum* | AU74 | MZ361634 | Aleta Wondo | Sidama | Semi forest | 64 |
| *T. asperellum* | AU75 | MZ361635 | Dale | Sidama | Garden | 74 |
| *T. asperellum* | AU76 | MZ361636 | Dale | Sidama | Garden | 64 |
| *T. orientale* | AU77 | MZ361637 | Dale | Sidama | Garden | 58 |
| *T. harzianum* | AU78 | MZ361629 | Gimbo | Kaffa | Forest | 70 |
| *T. viride* | AU79 |  | Gimbo | Kaffa | Forest | 64 |
| *T. asperellum* | AU80 |  | Haru | West Wollega | Forest | 64 |
| *T. asperellum* | AU81 | MZ361638 | Haru | West Wollega | Forest | 55.2 |
| *T. asperellum* | AU82 | MZ361639 | Sheko | Benchi Maji | Forest | 72 |
| *T. asperellum* | AU83 |  | Sheko | Benchi Maji | Forest | 56 |
| *T. harzianum* | AU84 | MZ361640 | Sheko | Benchi Maji | Forest | 60 |
| *T. asperelloides* | AU85 | MZ361641 | Sheko | Benchi Maji | Forest | 70 |
| *T. gamsii* | AU86 | MZ361642 | Sheko | Benchi Maji | Forest | 68 |
| *T. harzianum* | AU87 | MZ361643 | Gera | Jimma | Forest | 62.8 |
| *T. harzianum* | AU88 | MZ361644 | Gera | Jimma | Forest | 62 |
| *T. asperelloides* | AU89 |  | Gera | Jimma | Semi forest | 54 |
| *T. viride* | AU90 |  | Chena | Kaffa | Forest | 60.8 |
| *T. asperellum* | AU91 | MZ361645 | Chena | Kaffa | Forest | 68 |
| *T. brevicompactum* | AU92 |  | Chena | Kaffa | Forest | 68 |
| *T. reesei* | AU93 | MZ361646 | Chena | Kaffa | Semi forest | 54 |
| *T. aethiopicum* | AU94 | MZ361647 | Chena | Kaffa | Semi forest | 62 |
| *T. asperellum* | AU95 | MZ361648 | Limmu Saka | Jimma | Semi forest | 74.8 |
| *T. asperellum* | AU96 |  | Limmu Saka | Jimma | Semi forest | 65.2 |
| *T. asperellum* | **AU97** | **MZ361649** | **Limmu Saka** | **Jimma** | **Garden Coffee** | **79.3** |
| *T. asperelloides* | AU98 | MZ361650 | Limmu Saka | Jimma | Garden Coffee | 58 |
| *T. asperelloides* | AU99 | MZ361651 | Limmu Saka | Jimma | Garden Coffee | 74.6 |
| *T. asperellum* | AU100 | MZ361652 | Limmu Saka | Jimma | Garden Coffee | 72.8 |
| *Non identified* | AU101 |  | Limmu Saka | Jimma | Semi forest | 61.6 |
| *T. hamatum* | AU102 |  | Yeki | Shaka | Semi forest | 74.6 |
| *T. asperelloides* | AU103 | MZ361653 | Limmu Saka | Jimma | Semi forest | 62.8 |
| *T. asperellum* | AU104 | MZ361654 | Geisha | Kaffa | Forest | 62 |
| *T. harzianum* | **AU105** |  | **Geisha** | **Kaffa** | **Forest** | **78.7** |
| *T. aethiopicum* | **AU106** | **MZ361655** | **Geisha** | **Kaffa** | **Forest** | **79.3** |
| *Non identified* | AU107 |  | Geisha | Kaffa | Forest | 70 |
| *T. asperelloides* | AU108 | MZ361656 | Yeki | Shaka | Semi forest | 57.2 |
| *T. bissettii* | AU109 | MZ361657 | Yeki | Shaka | Semi forest | 65.6 |
| *T. asperellum* | AU110 | MZ361658 | Yeki | Shaka | Semi forest | 66 |
| *Non identified* | AU111 |  | Yeki | Shaka | Semi forest | 66 |
| *T. viride* | AU112 | MZ361659 | Yeki | Shaka | Garden Coffee | 60 |
| *T. asperellum* | AU113 |  | Yeki | Shaka | Garden Coffee | 60 |
| *T. longibrachiatum* | AU114 | MZ361660 | Yeki | Shaka | Garden Coffee | 52 |
| *T. asperellum* | AU115 | MZ361661 | Yeki | Shaka | Garden Coffee | 58 |
| *T. citrinoviride* | AU116 | MZ361662 | Yeki | Shaka | Semi forest | 64 |
| *Non identified* | AU117 |  | Yeki | Shaka | Semi forest | 68.8 |
| *T. asperelloides* | AU118 | MZ361663 | Limmu Saka | Jimma | Semi forest | 60 |
| *T. asperelloides* | AU119 |  | Yeki | Shaka | Semi forest | 60 |
| *T. asperelloides* | AU120 |  | Yeki | Shaka | Semi forest | 60 |
| *T. longibrachiatum* | AU121 |  | Sheko | Benchi Maji | Semi forest | 72.9 |
| *T. asperelloides* | AU122 | MZ361664 | Yeki | Shaka | Semi forest | 73.8 |
| *T. paratroviride* | AU123 | MZ361665 | Limmu Saka | Jimma | Semi forest | 68.8 |
| *T. asperelloides* | AU124 |  | Gera | Jimma | Semi forest | 66 |
| *T. longibrachiatum* | AU125 | MZ361666 | Yayu | Buno Bedele | Forest | 52 |
| *T. asperellum* | AU126 | MZ361667 | Yayu | Buno Bedele | Forest | 56 |
| *Non identified* | AU127 |  | Yayu | Buno Bedele | Forest | 56.8 |
| *T. asperellum* | AU128 |  | Yayu | Buno Bedele | Forest | 66 |
| *T. asperellum* | AU129 | MZ361668 | Yayu | Buno Bedele | Forest | 58 |
| *T. asperellum* | AU130 |  | Uraga | West Guji | Garden Coffee | 56 |
| *T. asperellum* | **AU131** | **MZ361669** | **Gera** | **Jimma** | **Forest Coffee** | **84.8** |
| *T. hamatum* | AU132 |  | Yirga cheffe | Gedeo | Garden Coffee | 70 |
| *T. asperellum* | AU133 | MZ361670 | Sheko | Benchi Maji | Semi forest | 52 |
| *T. asperelloides* | AU134 | MZ361671 | Sheko | Benchi Maji | Semi forest | 68 |
| *T. asperelloides* | AU135 | MZ361672 | Sheko | Benchi Maji | Semi forest | 66 |
| *T. longibrachiatum* | AU136 | MZ361673 | Sheko | Benchi Maji | Forest | 66.4 |
| *T. asperellum* | AU137 |  | Sheko | Benchi Maji | Forest | 62 |
| *T. longibrachiatum* | AU138 | MZ361674 | Semien Benchi | Benchi Maji | semi forest | 54.8 |
| *T. asperellum* | AU139 | MZ361675 | Semein Benchi | Benchi Maji | semi forest | 59.2 |
| *T. longibrachiatum* | AU140 |  | Semein Benchi | Benchi Maji | semi forest | 74.8 |
| *T. longibrachiatum* | AU141 | MZ361676 | Semein Benchi | Benchi Maji | semi forest | 74 |
| *T. asperellum* | AU142 |  | Sheko | Benchi Maji | semi forest | 70 |
| *T. longibrachiatum* | AU143 | MZ361677 | Sheko | Benchi Maji | semi forest | 62 |
| *T. asperelloides* | AU144 | MZ361678 | Sheko | Benchi Maji | Forest | 74.6 |
| *T. reesei* | AU145 | MZ361679 | Sheko | Benchi Maji | Forest | 68 |
| *T. asperelloides* | AU146 |  | Sheko | Benchi Maji | Forest | 64 |
| *Non identified* | AU147 |  | Sheko | Benchi Maji | Forest | 62 |
| *T. asperelloides* | AU148 | MZ361680 | Haru | West Wollega | Forest | 64 |
| *T. asperelloides* | AU149 | MZ361681 | Haru | West Wollega | Semi forest | 52 |
| *T. gamsii* | AU150 | MZ361682 | Haru | West Wollega | Semi forest | 64 |
| *Non identified* | AU151 |  | Haru | West Wollega | Semi forest | 68 |
| *T. longibrachiatum* | AU152 |  | Limmu Saka | Jimma | Semi forest | 74.8 |
| *T. asperellum* | AU153 |  | Delo Mena | Bale | Forest | 54 |
| *T. harzianum* | AU154 |  | Delo Mena | Bale | Forest | 70 |
| *T. asperelloides* | AU155 | MZ361683 | Delo Mena | Bale | Forest | 70 |
| *T. asperelloides* | AU156 |  | Delo Mena | Bale | Forest | 58 |
| *T. aethiopicum* | AU157 |  | Berbere | Bale | Forest | 62.8 |
| *T. longibrachiatum* | **AU158** | **MZ361684** | **Yeki** | **Sheka** | **Forest** | **82.4** |
| *T. brevicompactum* | AU159 |  | Berbere | Bale | Forest | 60 |
| *T. gamsii* | AU160 |  | Berbere | Bale | Forest | 58 |
| *T. longibrachiatum* | AU161 | MZ361685 | Berbere | Bale | Forest | 50 |
| *T. asperelloides* | AU162 | MZ361686 | Kercha | West Guji | Garden Coffee | 66 |
| *T. asperellum* | AU163 |  | Jarso | West Wollega | Semi forest | 50 |
| *T. longibrachiatum* | AU164 | MZ361687 | Jarso | West Wollega | Semi forest | 54.8 |
| *T. asperellum* | AU165 | MZ361688 | Aira Guliso | West Wollega | Semi forest | 56 |
| *T. hamatum* | AU166 | MZ361689 | Semein Benchi | Benchi Maji | Semi forest | 56 |
| *T. bissettii* | AU167 | MZ361690 | Bule Hora | West Guji | Garden Coffee | 66 |
| *T. aethiopicum* | AU168 |  | Kercha | West Guji | Garden Coffee | 68 |
| *T. asperellum* | AU169 | MZ361691 | Kercha | West Guji | Garden Coffee | 74.8 |
| *T. asperellum* | AU170 |  | Aira Guliso | West Wollega | Garden Coffee | 74.84 |
| *T. asperellum* | **AU171** | **MZ361692** | **Aira Guliso** | **West Wollega** | **Garden Coffee** | **77.7** |
| *T. asperellum* | AU172 | MZ361693 | Kercha | West Guji | Garden Coffee | 72.3 |
| *T. longibrachiatum* | AU173 | MZ361694 | Bule Hora | West Guji | Garden Coffee | 74.8 |
| *T. asperellum* | AU174 | MZ361695 | Bule Hora | West Guji | Garden Coffee | 73.8 |
| *T. asperelloides* | AU175 | MZ361692 | Bedele | Buno Bedele | Forest | 73.4 |
